# EPHX1 enhances drug resistance to regorafenib by activating the JAK/STAT signaling pathway in hepatocellular carcinoma cell lines

**DOI:** 10.1186/s41065-025-00517-1

**Published:** 2025-07-31

**Authors:** Bin Xu, Xiangnan Liang, Wuguang Liu, BaiTong Wu, Qiuxiang Wang, Gong Kai, Chun Han, Binwen Sun, Bing Dong, Chengyong Dong, Liming Wang

**Affiliations:** 1 Engineering Research Center for New Materials and Precision Treatment Technology of Malignant Tumors Therapy, The Second Affiliated Hospital, Dalian Medical University, Dalian, Liaoning 116027 China; 2 Engineering Technology Research Center for Translational Medicine, The Second Affiliated Hospital, Dalian Medical University, Dalian, Liaoning 116027 China; 3 Division of Hepatobiliary and Pancreatic Surgery, Department of General Surgery, The Second Affiliated Hospital of Dalian Medical University, 467 Zhongshan Road, Dalian, Liaoning 116027 China; 4 Nephrology Department, The Second Affiliated Hospital of Dalian Medical University, Dalian, Liaoning, 116000 China; 5 Laboratory of Immune and Metabolic Kidney Diseases, The Second Affiliated Hospital of Dalian Medical University, 467 Zhongshan Road, Dalian, Liaoning, 116023 China

**Keywords:** EPHX1, Hepatocellular carcinoma, Regorafenib, Drug resistance, JAK/STAT pathway

## Abstract

**Background:**

Regorafenib serves as a second-line treatment for patients with advanced hepatocellular carcinoma (HCC). Microsomal epoxide hydrolase 1 (EPHX1) is closely associated with tumorigenesis and drug resistance. However, the relationship between EPHX1 and regorafenib resistance, as well as the underlying mechanisms in HCC, remains unclear.

**Objective:**

To investigate the role and mechanisms of EPHX1 in mediating regorafenib resistance in HCC.

**Methods:**

We assessed the protein expression levels of EPHX1 in human HCC tissues and adjacent non-tumor tissues. Subsequently, we constructed HCC cell lines with EPHX1 overexpression and knockdown using lentiviral vectors and stimulated these cells with varying concentrations of regorafenib. We then measured cell proliferation and apoptosis using flow cytometry and Western blotting. Additionally, we established xenograft tumor models to explore the impact of EPHX1 on the in vivo efficacy of regorafenib. Furthermore, we employed digital gene expression sequencing (DGE-seq) to investigate and validate the specific molecular mechanisms by which EPHX1 mediates regorafenib resistance in HCC cells.

**Results:**

We found that EPHX1 protein levels were significantly higher in HCC tissues compared to adjacent non-tumor tissues. EPHX1 inhibited the effects of regorafenib on cell proliferation and apoptosis. Consistently, the efficacy of regorafenib was enhanced in vivo following EPHX1 knockdown. Moreover, KEGG pathway enrichment analysis of DGE-seq data indicated that the JAK/STAT signaling pathway is crucial for EPHX1-induced regorafenib resistance. Finally, EPHX1 suppressed regorafenib-induced inactivation of the JAK/STAT signaling pathway and blocking this pathway with HY-N1447 alleviated EPHX1-induced regorafenib resistance.

**Conclusion:**

In summary, we conclude that EPHX1 enhances regorafenib resistance in HCC by activating the JAK/STAT signaling pathway. Our findings suggest that EPHX1 is a key resistance-related gene, which has significant implications for the application of regorafenib in advanced HCC.

**Supplementary Information:**

The online version contains supplementary material available at 10.1186/s41065-025-00517-1.

## Introduction

Liver cancer is the sixth most common cancer globally, with approximately 860,000 new cases annually, accounting for about 4.3% of all cancers worldwide. It is also the fourth leading cause of cancer-related deaths, with around 750,000 deaths annually, representing 7.8% of all cancer-related deaths [1]. Hepatocellular carcinoma (HCC) constitutes about 90% of all liver cancer cases [2].

Most HCC patients are diagnosed at advanced stages, limiting treatment options such as surgical resection and liver transplantation [3]. Over half of HCC patients receive systemic therapy [4]. According to FDA approvals, sorafenib, Lenvatinib, and the combination of atezolizumab with bevacizumab are recommended as first-line treatments for HCC [5]. Recent Chinese guidelines have added regorafenib as a second-line systemic treatment for patients who progress after first-line therapy [6]. Regorafenib, typically used as a second-line treatment, provides additional therapeutic options and significantly extends progression-free survival (PFS) and overall survival (OS) [7]. Recent studies suggest that the combination of pembrolizumab and regorafenib shows potential as a first-line treatment. Despite its limited survival benefits, regorafenib remains an effective treatment for HCC patients with impaired liver function [8]. However, the therapeutic efficacy of regorafenib is constrained by drug resistance [9], the mechanisms of which remain unclear [10]. Therefore, further research into the mechanisms of regorafenib resistance is urgently needed, and this study may also explore new strategies for HCC drug therapy.

Microsomal epoxide hydrolase 1 (EPHX1), located on chromosome 1, consists of nine exons and has a molecular weight of 35 kDa [11]. It is primarily distributed in the endoplasmic reticulum of eukaryotic cells and is expressed in almost all tissues [12]. Previous studies have shown that EPHX1 polymorphisms are closely associated with various diseases, including esophageal cancer [13], lung cancer [14], head and neck cancer [15], colorectal cancer [16], ovarian cancer [17], and liver cancer [18]. EPHX1 plays a crucial role in biotransformation, metabolism [19], and drug metabolism [20, 21].

However, the relationship between EPHX1 expression and chemotherapy resistance in HCC remains unclear. Therefore, our study aims to elucidate the relationship between EPHX1 and regorafenib resistance in HCC and further explore the specific molecular mechanisms underlying regorafenib resistance.

## Materials and methods

### Patients and tissue samples

Surgical resection samples from 15 HCC patients, including HCC tissues and paired adjacent non-tumor tissues, were obtained from the Second Hospital of Dalian Medical University. The samples were immediately frozen at -80 °C until protein extraction. This study adhered to the Declaration of Helsinki and was approved by the hospital’s ethics committee. All patients provided informed consent, and HCC diagnosis was confirmed by histopathological examination post-surgery. None of the patients had undergone chemotherapy or radiotherapy prior to surgery.

### Immunohistochemical analysis

Human HCC and adjacent non-tumor tissues were fixed with 4% paraformaldehyde, embedded in paraffin, and sectioned. Immunohistochemical (IHC) staining was performed using EPHX1 antibody (dilution 1:2000; catalog number: DF6799; RRID: AB_2838761; Affinity Biosciences Ltd.). Images were captured using an upright microscope, and the percentage of positive areas in the micrographs was quantified using Case Viewer (Just Systems, Japan).

### Cell culture

Human HCC cell lines (SNU449, Huh7, SNU387, MHCC97-H) were obtained from the Cell Bank/Stem Cell Bank of the Chinese Academy of Sciences (Shanghai, China). All cells were cultured in Dulbecco’s Modified Eagle Medium (DMEM, Hyclone, South Logan, UT, USA) supplemented with 10% fetal bovine serum (FBS, Gibco BRL, Grand Island, NY, USA) and 1% penicillin/streptomycin (100 U/ml, Gibco BRL, Grand Island, NY, USA). Cells were maintained at 37 °C in a 5% CO2 atmosphere. EPHX1 overexpression was achieved by transfecting cells with lentiviral vectors carrying EPHX1 (OE-EPHX1) for 72 h (Shanghai GeneChem Co., Ltd., Shanghai, China). EPHX1 knockdown was achieved by transfecting cells with lentiviral vectors carrying EPHX1-specific shRNA (sh-EPHX1) for 72 h (Shanghai Gene Pharma Co., Ltd., Shanghai, China). HCC cells were co-incubated with regorafenib (MedChemExpress, Monmouth Junction, NJ, USA) for 24–48 h. HY-N1447 (an inhibitor of the JAK/STAT signaling pathway, 20 µM, 24 h) (MedChemExpress, Monmouth Junction, NJ, USA) was used in combination with regorafenib in EPHX1-overexpressing cells.

### Western blot

Cells or tissues were lysed in radioimmunoprecipitation assay (RIPA) buffer (Keygen Biotech Co., Ltd., Nanjing, China) supplemented with 1 µL phenylmethylsulphonyl fluoride (PMSF, Merck Millipore, Billerica, MA, USA) and 1 µL phosphatase inhibitor (Transgene Biotech, Beijing, China) per 100 µL buffer. Protein concentration was determined using a BCA protein assay kit (Keygen Biotech Co., Ltd., Nanjing, China). Proteins were denatured by boiling in SDS loading buffer (New Cell & Molecular Biotech Co., Ltd., Suzhou, China) for 10 min. Equal amounts of protein were separated by SDS-PAGE (Epizyme Biotechnology Co., Ltd., Shanghai, China) and transferred to polyvinylidene fluoride (PVDF) membranes (Merck Millipore, Billerica, MA, USA). Protein bands were detected using an ECL chemiluminescence kit (Yeasen Biotechnology (Shanghai) Co., Ltd., Shanghai, China) and normalized to GAPDH. Primary antibodies used included EPHX1 (dilution 1:2000; catalog number: DF6799; RRID: AB_2838761; Affinity Biosciences Ltd.), PCNA (dilution 1:10000; catalog number: p12004; RRID: AB_2160330; Proteintech Group, Inc.), Bax (dilution 1:5000; catalog number: O07812; RRID: AB_2061561; Proteintech Group, Inc.), Bcl2 (dilution 1:2000; catalog number: P10415; RRID: AB_2227948), Caspase3 (dilution 1:5000; catalog number: P42574; RRID: AB_10733244), JAK1 (dilution 1:500; catalog number: GB115719-100), p-JAK (dilution 1:200; catalog number: GB115604-100), JAK2 (dilution 1:750; catalog number: GB11325-100), p-JAK2 (dilution 1:400; catalog number: GB114585-100), STAT3 (dilution 1:2000; catalog number: BC000727, RRID: AB_2302876), and p-STAT3 (dilution 1:100; catalog number: GB150001-100).

### Cell viability assay

Cell viability was assessed using the Cell Counting Kit-8 (CCK-8) assay (MedChem Express, Monmouth Junction, NJ, USA) to determine the half-maximal inhibitory concentration (IC50). Cells were seeded at a density of 2 × 10^3 cells per well in 96-well plates and incubated with varying concentrations of regorafenib. Subsequently, 110 µL of serum-free medium mixed with CCK-8 solution (10:1 ratio) was added to each well, and cells were incubated at 37 °C for 2 h. Absorbance (OD) was measured at 450 nm using a microplate reader (Molecular Devices, USA).

### Colony formation assay

Cells were seeded at a density of 800 cells per well in 6-well plates, with three replicates per group. Cells were treated with varying concentrations of regorafenib, and the medium was replaced every three days for 14 days. Colonies were washed three times with phosphate-buffered saline (PBS) (Seven Innovation Beijing Biotechnology Co., Ltd., Beijing, China), fixed with 4% paraformaldehyde for 20 min, and stained with crystal violet (Beijing Solarbio Science & Technology Co., Ltd., Beijing, China) for 20 min at room temperature. After drying, colonies were counted using ImageJ software.

### Cell cycle analysis

Cells were cultured in 6-well plates and treated with varying concentrations of regorafenib for 24 h. Cells were collected, washed with pre-chilled PBS, and resuspended in pre-chilled DEPC water (Seven Innovation Beijing Biotechnology Co., Ltd., Beijing, China). Cells were then fixed in pre-chilled absolute ethanol (Tianjin Tianli Chemical Reagent Co., Ltd., Tianjin, China) (DEPC water: ethanol = 1:3) at 4 °C for 12 h and stained with PI/RNase staining solution (Keygen Biotech Co., Ltd., Nanjing, China) at 37 °C in the dark for 30 min. Samples were analyzed using a flow cytometer (BD Biosciences, San Jose, CA, USA).

### Apoptosis assay

Cells were collected and washed with pre-chilled PBS, and apoptosis was detected using the Annexin V Apoptosis Detection Kit (Elabscience Biotechnology Co., Ltd., Wuhan, China). Cells were stained with 2.5 µL Annexin V-APC, 2.5 µL propidium iodide (PI), and 500 µL binding buffer in the dark at room temperature for 20 min. Samples were analyzed using a FACSVerse flow cytometer.

### Quantitative real-time PCR

Total RNA was extracted using the TRIzol reagent kit (Seven Innovation Beijing Biotechnology Co., Ltd., Beijing, China). RNA concentration was measured using a Nanodrop 2000 C, and cDNA was synthesized using the Prime Script RT reagent kit (Beijing Transgene Biotech Co., Ltd., Beijing, China). Quantitative PCR was performed using the Perfect Start Green qPCR Super Mix PCR kit (Beijing Transgene Biotech Co., Ltd., Beijing, China) on a real-time PCR machine (Thermos Fisher Scientific, Waltham, MA, USA). Relative mRNA expression levels were normalized to GAPDH. Primers were synthesized by Gene Pharma (Shanghai Gene Pharma Co., Ltd., Shanghai, China). The forward and reverse primers for EPHX1 were 5’-CATGAGAAGTATGACAACAGCCT-3’ and 5’-AGTCCTTCCACGATACCAAAGT-3’, respectively. The forward and reverse primers for GAPDH were 5’-GACCTGCTGACCAACGTCAT-3’ and 5’-GAGAAGCCAGTGGGCACATA-3’, respectively.

### Animal experiments

BALB/c nude mice were purchased from Beijing Vital River Laboratory Animal Technology Co., Ltd. Huh7 cells (2 × 10^6) transfected with sh-EPHX1, sh-Con, OE-EPHX1, or NC-EPHX1 were subcutaneously injected into 4-week-old female BALB/c nude mice. Tumor size was measured every three days, and tumor volume was calculated using the formula (length × width^2)/2. When tumor volume reached approximately 100 mm^3, mice were randomly divided into groups (*n* = 5 per group). Mice in the sh-Con + Regorafenib, sh-EPHX1 + Regorafenib, OE-EPHX1 + Regorafenib, and NC-EPHX1 + Regorafenib groups were administered regorafenib (30 mg/kg/d) by oral gavage for 14 days. At the end of the experiment, mice were euthanized, and tumors were excised, photographed, and weighed.

### Digital gene expression profiling

Two groups of SNU449 cells with stable EPHX1 knockdown and treated with regorafenib (sh-Con + Regorafenib and sh-EPHX1 + Regorafenib) were used for digital gene expression (DGE) sequencing analysis, with three replicates per group. Samples were sent to Wuhan Met ware Biotechnology Co., Ltd. (Wuhan, China) for DGE sequencing. The experimental workflow included RNA extraction, RNA quality control, library construction, and sequencing. Differential gene expression analysis and functional enrichment analysis were performed. Differentially expressed genes were selected based on|log2Fold Change| ≥ 1 and FDR < 0.05.

### Statistical analysis

Data were analyzed using GraphPad Prism 9 (ANOVA, t-test, chi-square test), and quantitative data plots were generated automatically.

### Illustrations

Illustrations in the schematic diagrams were provided by Fig draw.

## Results

### Upregulation of EPHX1 in HCC tissues

**Fig. 1:**
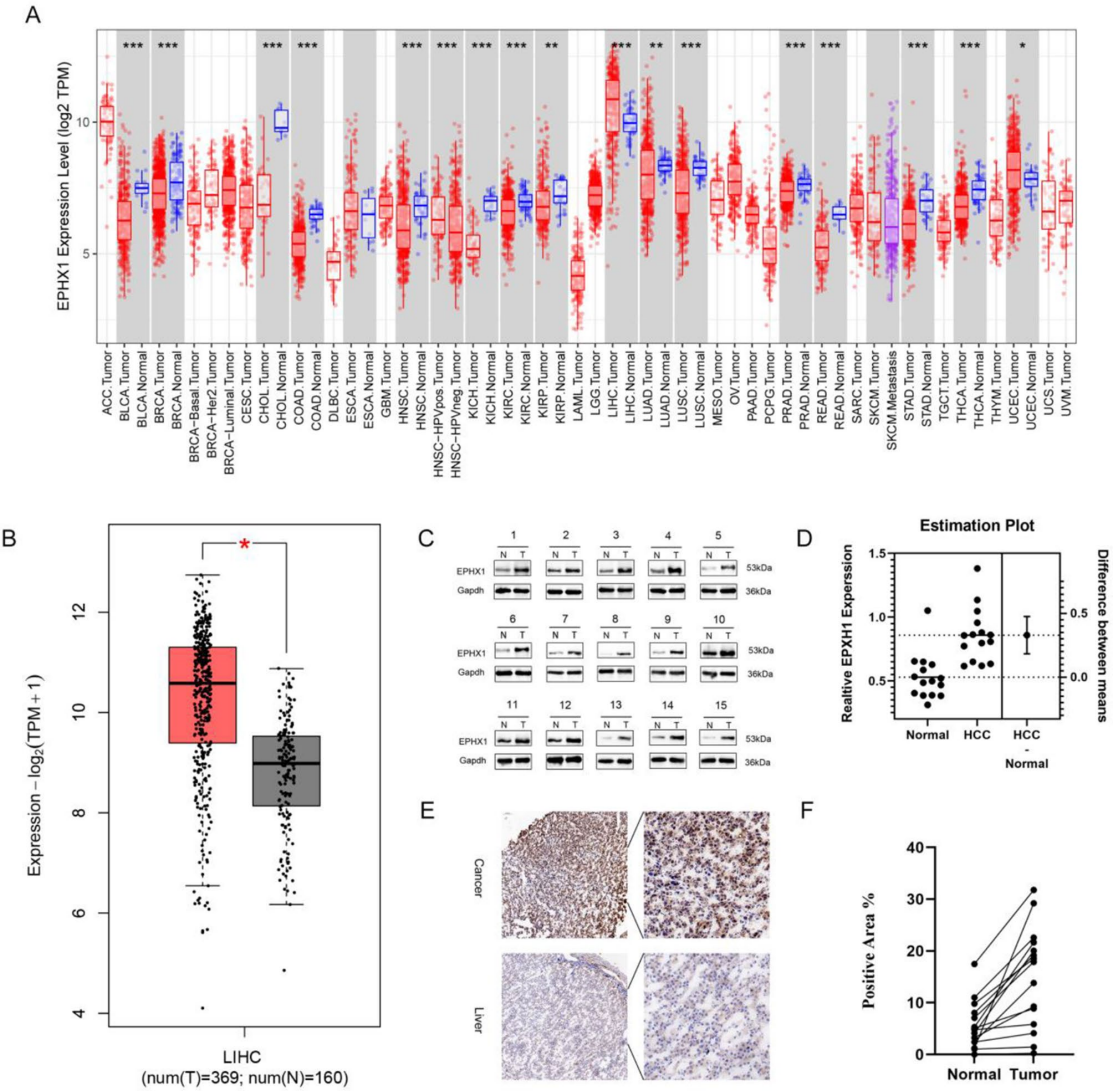
Increased expression of EPHX1 in patients with hepatocellular carcinoma: (**A**) A pan-cancer analysis of EPHX1 expression in the TIMER2.0 database. (**B**) Differences in expression of EPHX1 in 529 tumor and normal samples in the database (LIHC: Liver hepatocellular carcinoma; image information from GEPIA). (**C**) Western blot analysis was performed to detect the expression of EPHX1 in 15 pairs of hepatocellular carcinoma (HCC) tissues and corresponding non-tumor tissues. (**D**) Quantitative analysis of EPHX1 protein levels is shown. (**E**) Paraffin-embedded liver sections were stained with EPHX1 immunohistochemistry. (**F**) Quantitative analysis showing EPHX1 immunohistochemical levels

To investigate the correlation between EPHX1 expression in hepatocellular carcinoma (HCC) patients, we conducted bioinformatics analyses using the TIMER2.0 and GEPIA databases (http://gepia.cancer-pku.cn). Pan-cancer analysis revealed significantly elevated EPHX1 expression in tumor tissues compared to normal counterparts (Fig. 1A). Specifically, in HCC samples, both mRNA and protein levels of EPHX1 were markedly higher than in adjacent non-tumor tissues (Fig. 1B). To validate these findings, we performed Western blot analysis on 15 paired HCC and paracancerous tissues. Consistent with database results, EPHX1 protein expression was significantly upregulated in all 15 HCC specimens (Fig. 1C-D). Further immunohistochemical (IHC) analysis of paraffin-embedded HCC tissues confirmed cytoplasmic localization of EPHX1 with stronger immunoreactivity in tumor tissues (Fig. 1E-F). Collectively, these results demonstrate that EPHX1 is aberrantly overexpressed in HCC, suggesting its potential role in tumor progression.

### EPHX1 confers regorafenib resistance in HCC cells

**Fig. 2:**
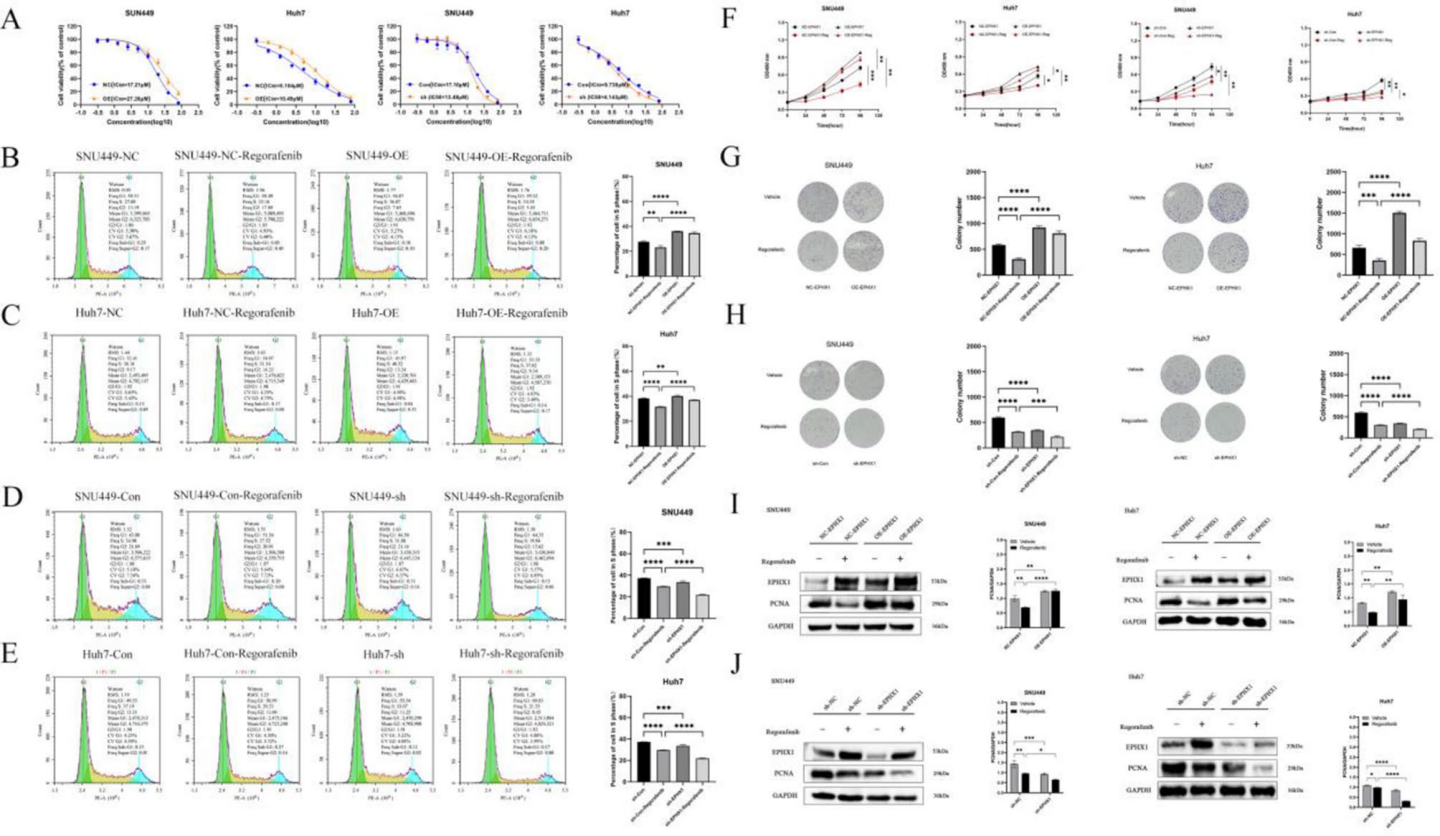
EPHX1 antagonizes Regorafenib-induced growth suppression in HCC cells. (**A**) The IC50 values of Regorafenib in SNU449 and Huh7 cells with the NC-EPHX1 and OE-EPHX1 were detected by the Cell Counting Kit-8 (CCK-8) assay. (**B**-**E**) Flow cytometry was conducted to perform cell cycle distribution analysis of the cells in NC-EPHX1 ± Regorafenib, OE-EPHX1 ± Regorafenib, Con-EPHX1 ± Regorafenib and sh-EPHX1 ± Regorafenib groups of SNU449 and Huh7 cells. (**F**) Growth curves measured by CCK8 showed the growth rates in NC-EPHX1, OE-EPHX1, sh-Con and sh-EPHX1 groups of SNU449 and Huh7 cells. (**G**-**H**) Representative images of colonies formed in SNU449 and Huh7 cells determined by plate cloning experiments. (**I**-**J**) Western blot analysis showed proliferating cell nuclear antigen (PCNA) protein expression (normalized to (GAPDH) in NC-EPHX1 ± Regorafenib, OE-EPHX1 ± Regorafenib, sh-Con ± Regorafenib and sh-EPHX1 ± Regorafenib groups of SNU449 and Huh7 cells. *p < 0.05, **p < 0.01, ***p < 0.001, ****p < 0.0001

Emerging evidence implicates EPHX1 in chemoresistance across multiple malignancies. We first determined regorafenib IC50 values in four HCC cell lines: SNU449 (17.04 µM), SNU387 (8.865 µM), MHCC97-H (8.056 µM), and Huh7 (5.210 µM) (Supplementary Figure S1A). Notably, 24-hour regorafenib treatment upregulated EPHX1 expression in all tested cell lines (Supplementary Figure S1B-C). Using lentiviral transduction, we established stable EPHX1-overexpressing (OE-EPHX1) and knockdown (sh-EPHX1) cell lines with respective controls (Supplementary Figure S2). Subsequent IC50 assays revealed that EPHX1 overexpression increased regorafenib resistance (elevated IC50), whereas EPHX1 silencing enhanced drug sensitivity (reduced IC50) in both SNU449 and Huh7 cells (Fig. 2A). Cell cycle analysis demonstrated that regorafenib treatment reduced S-phase cell populations, an effect counteracted by EPHX1 overexpression (Fig. 2B-C). Conversely, EPHX1 knockdown potentiated regorafenib-induced S-phase suppression (Fig. 2D-E). Complementary assays including CCK-8 proliferation (Fig. 2F), colony formation (Fig. 2G-H), and PCNA expression analysis (Fig. 2I-J) consistently indicated that EPHX1 promotes regorafenib resistance by sustaining proliferative capacity.

**Fig. 3:**
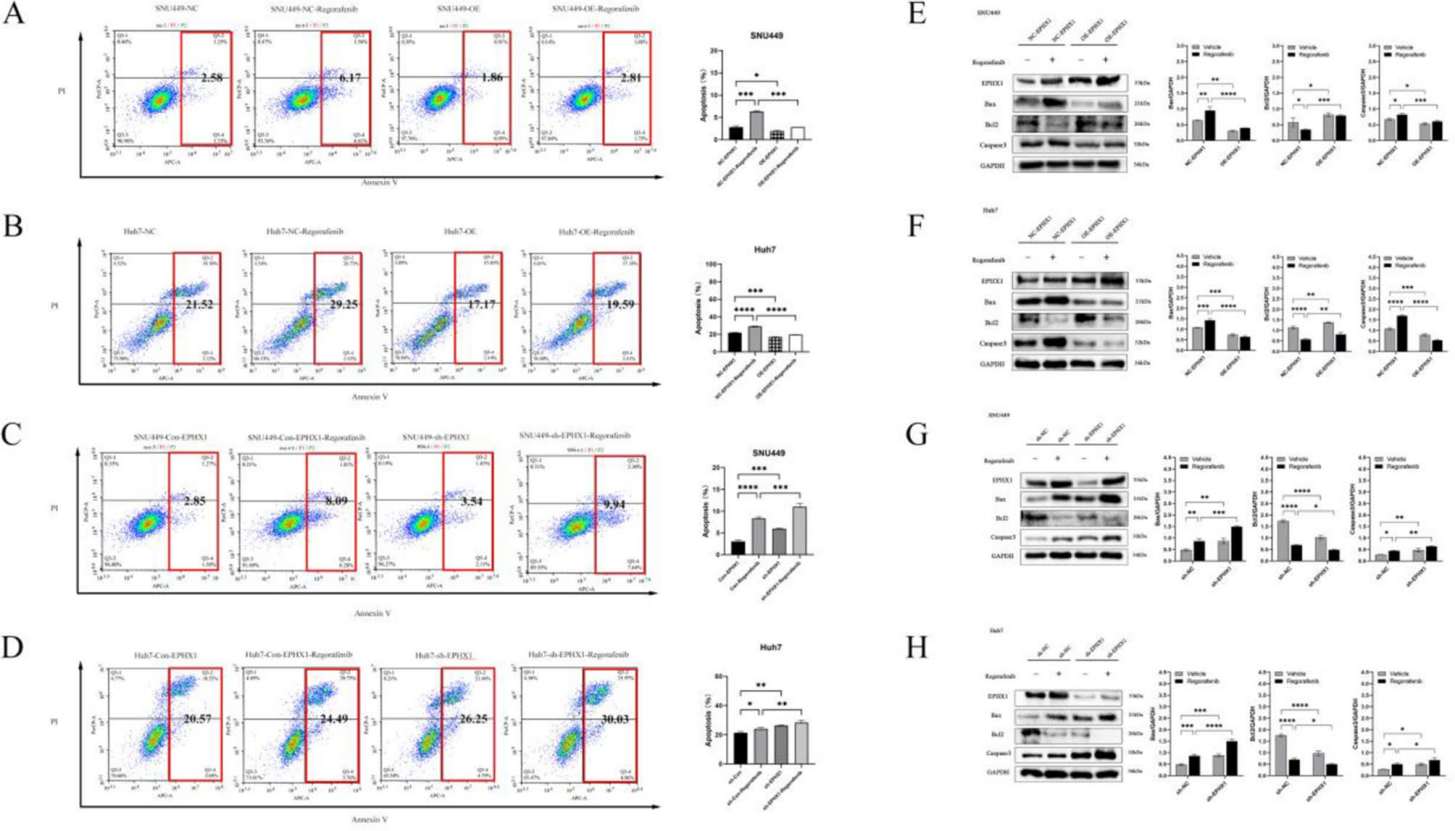
EPHX1 blocks Regorafenib-induced apoptosis in HCC cells. (**A**-**D**) The amount of apoptosis of NC-EPHX1±Regorafenib, OE-EPHX1±Regorafenib, sh-Con±Regorafenib and sh-EPHX1±Regorafenib in SNU449 and Huh7 cells were detected by flow cytometry. (**E**-**H**) Western blots were used to detect the expression of Bax, Bcl2 and Caspase-3 in NC-EPHX1±Regorafenib, OE-EPHX1±Regorafenib, sh-Con±Regorafenib and sh-EPHX1±Regorafenib in SNU449 and Huh7 cells, *p < 0.05, **p < 0.01. ***p < 0.001.

Flow cytometry revealed enhanced apoptosis induction by regorafenib, which was attenuated in EPHX1-overexpressing cells (Fig. 3A-B) and augmented in EPHX1-knockdown counterparts (Fig. 3C-D). Western blot analysis of apoptosis-related proteins showed regorafenib upregulated pro-apoptotic Bax and cleaved caspase-3 while downregulating anti-apoptotic Bcl-2. These effects were reversed by EPHX1 overexpression (Fig. 3E-F) and potentiated by EPHX1 silencing (Fig. 3G-H).

### EPHX1 knockdown enhances regorafenib efficacy in vivo

**Fig. 4:**
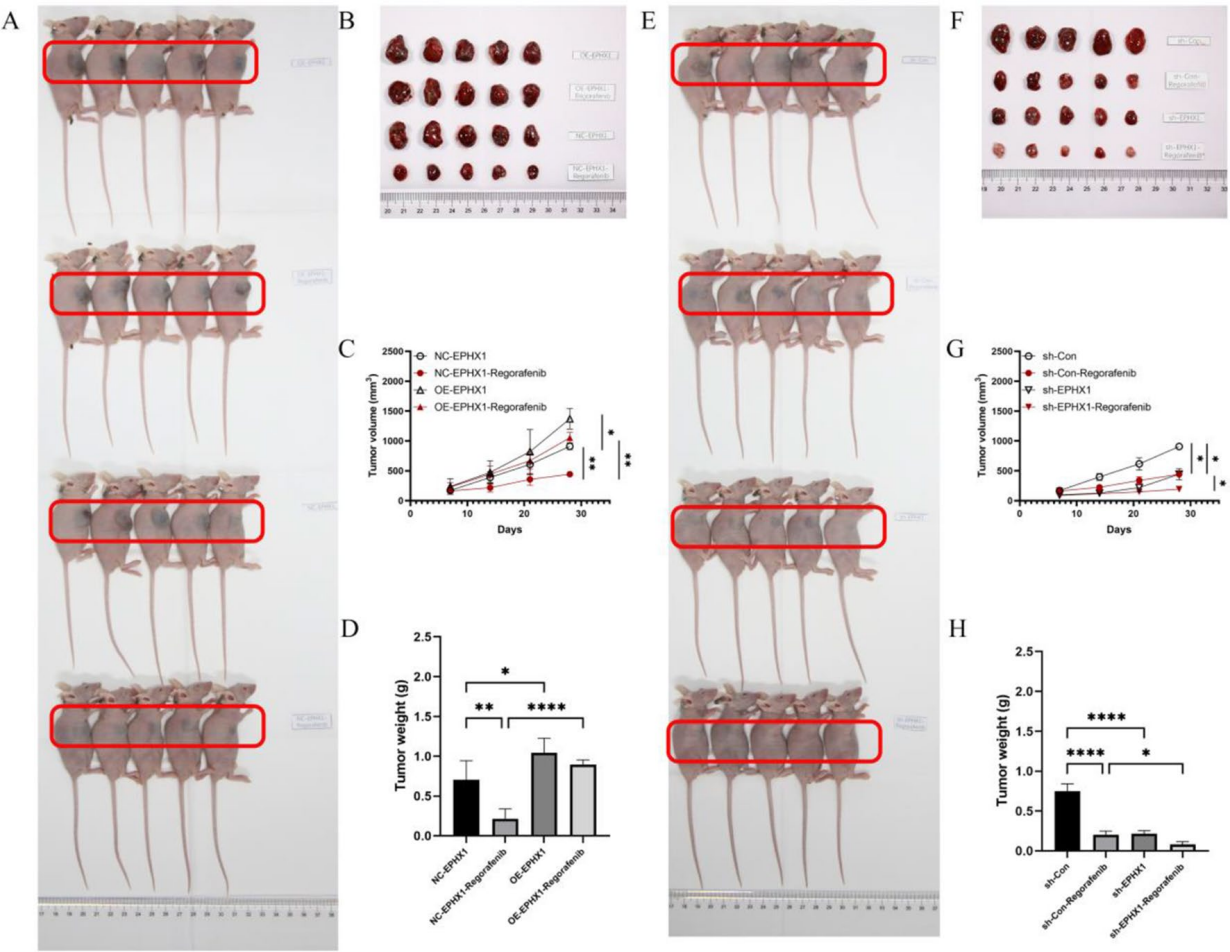
Regorafenib efficiency after EPHX1 knockdown or overexpression in vivo. (**A**-**B**) The morphologies of collected tumors in subcutaneous NC-EPHX1 ± Regorafenib vs. OE-EPHX1 ± Regorafenib xenografts in nude mice. (**C**) Tumor growth curves. Tumor weights were measured after collection. (**E**-**F**) The morphologies of collected tumors in subcutaneous sh-Con + Regorafenib vs. sh-EPHX1 ± Regorafenib xenografts in nude mice. (**G**) Tumor growth curves. (**H**): Tumor weights were measured after collection, * *p* < 0.05, ** *p* < 0.01. *** *p* < 0.001

In a xenograft model using Huh7 cells with stable EPHX1 knockdown, both EPHX1 depletion and regorafenib monotherapy inhibited tumor growth, with maximal suppression achieved by combination therapy (Fig. 4A-B). Tumor weight analysis corroborated the synergistic effect of EPHX1 knockdown and regorafenib treatment (Fig. 4C).

### EPHX1 mediates resistance via JAK1/JAK2/STAT3 activation

**Fig. 5:**
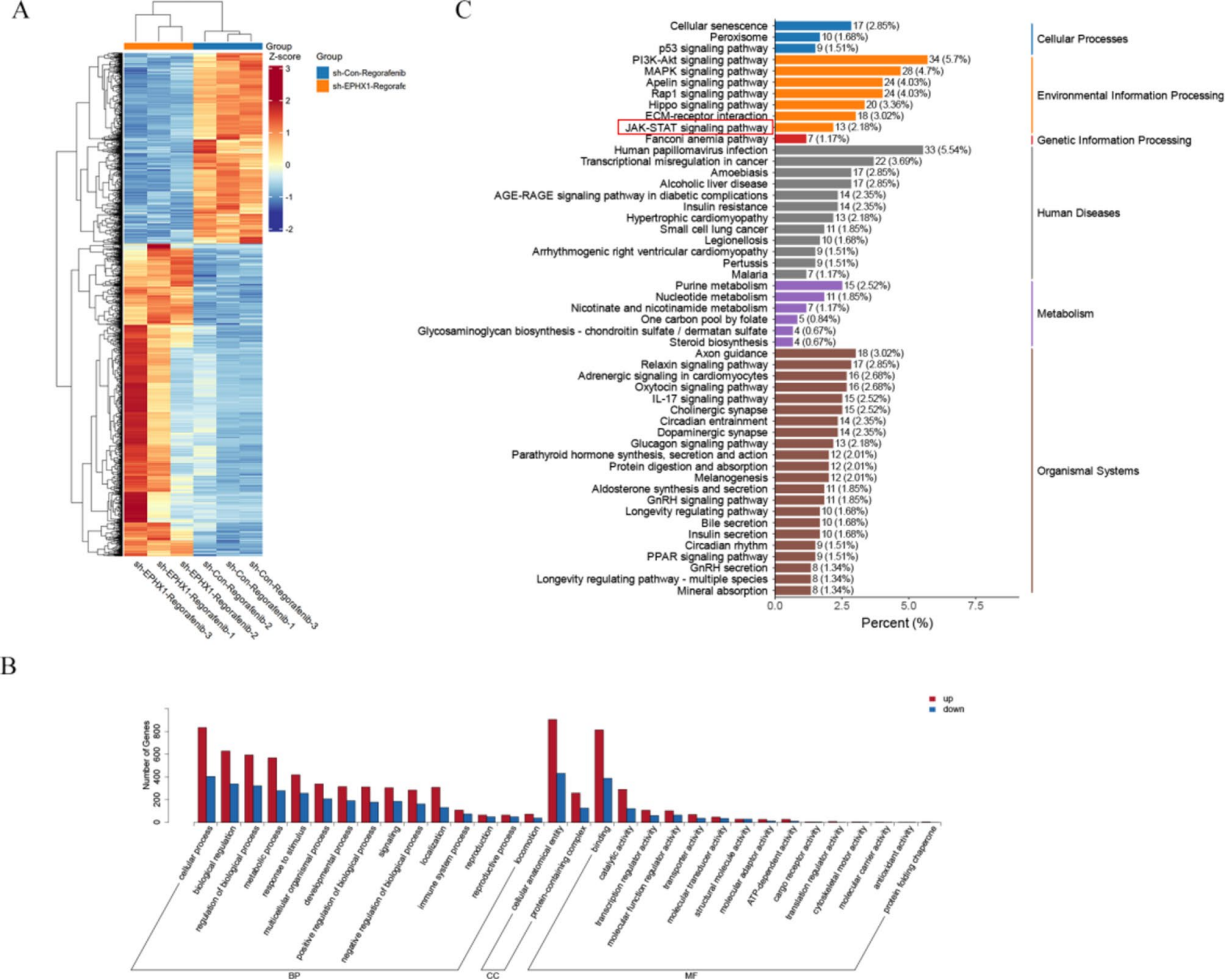
Digital gene expression profiling-seq analysis in SNU449 cells. (**A**) Heat map of the results from the cluster analysis of the digital gene expression profiling (DGE)-seq data (sh-Con + Regorafenib vs. sh-EPHX1 + Regorafenib). Each column denotes a sample. Each row indicates a DEG (differentially expressed gene). For each gene, red represents a high level of expression relative to the mean, while blue expresses a low level. The scale bar is the number of standard deviations from the mean. (**B**) Bar chart of GO functional analysis for the DEGs obtained from DGE sequencing. GO terms with *p* < 0.05 were thought to be notably enriched by DEGs. The y axis represents the number of genes in a GO classification category. Red represents increased expression in the shEPHX1 + Regorafenib group, whereas blue indicates decreased expression in that group. (**C**) Pathway enrichment analysis showed the top 20 signaling pathways in KEGG (*p* < 0.05)

**Fig. 6:**
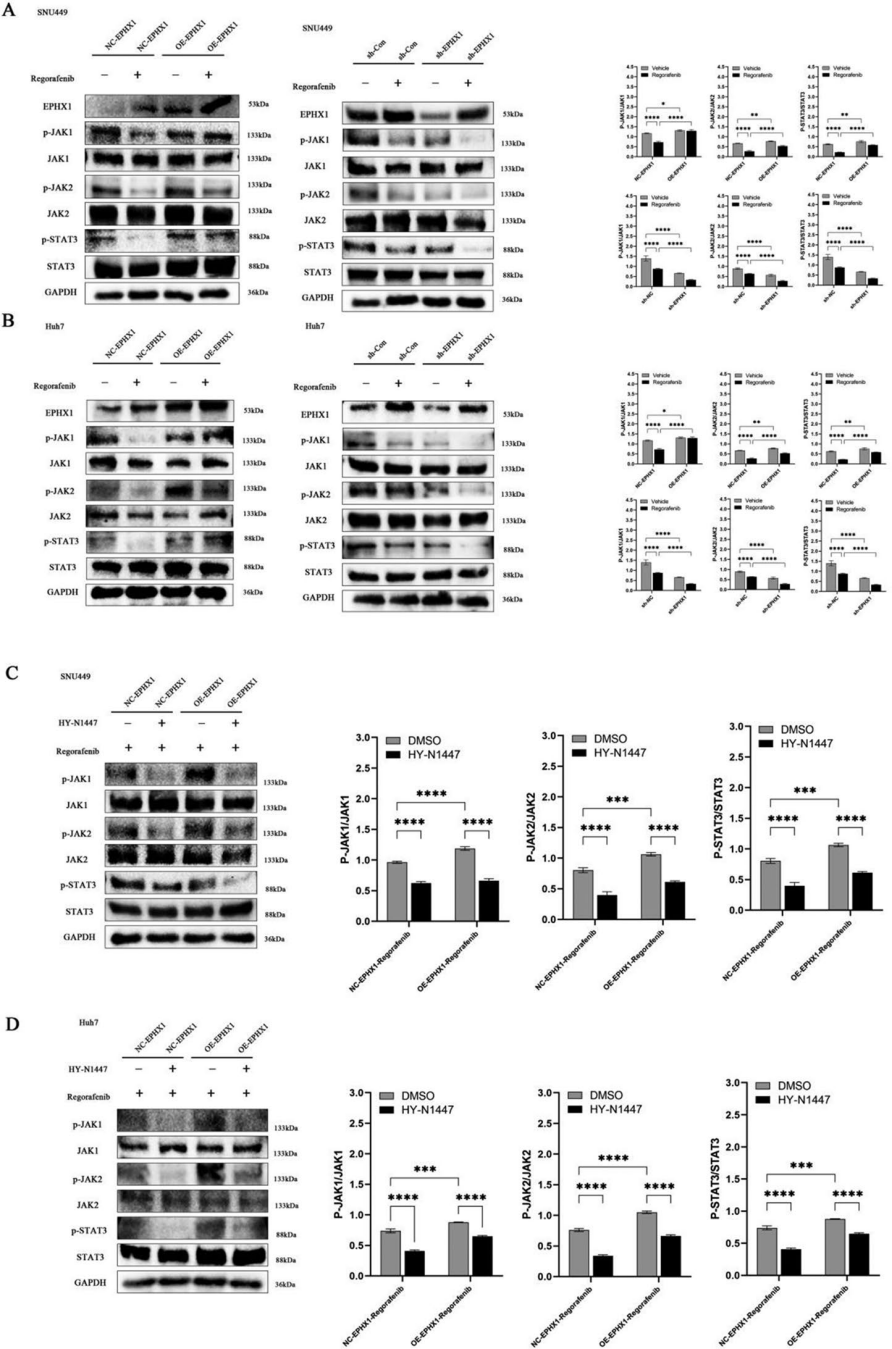
EPHX1 antagonizes Regorafenib-mediated JAK1-JAK2-STAT3 signaling pathway inhibition. (**A**-**B**) The protein expression levels of Janus Kinase 1(JAK1), phosphorylated Janus Kinase 1(p-JAK1), phosphoinositide Janus Kinase 2(JAK2), Janus Kinase 2(p-JAK2), signal Transducer And Activator Of Transcription 3 (STAT3) and phosphoinositide signal Transducer And Activator Of Transcription 3 (p-STAT3) were detected by Western blot analysis in cells infected NC-EPHX1 ± Regorafenib vs. cells infected OE-EPHX1 ± Regorafenib groups and sh-Con ± Regorafenib vs. cells infected sh-EPHX1 ± Regorafenib groups of SNU449 and Huh7 cells. (**C**-**D**) Western blot analysis was also applied to evaluate the expression of p-JAK1, JAK1, p-JAK2, JAK2, p-STAT3 and STAT3 in the NC-EPHX1 + Regorafenib, NC-EPHX1 + Regorafenib + HY-N1447, sh-Con + Regorafenib, sh-EPHX1 + Regorafenib-HY-N1447 groups of SUN449 and Huh7 cells. Protein samples derived from the same experiment and gels were processed in parallel, * *p* < 0.05, ** *p* < 0.01. *** *p* < 0.001

RNA sequencing of SNU449 cells treated with regorafenib (sh-NC vs. sh-EPHX1) identified significant alterations in the JAK/STAT signaling pathway through KEGG enrichment analysis (Fig. 5). Given the established relevance of JAK/STAT in HCC pathogenesis and regorafenib response, we further investigated this association. Concomitant use of the JAK/STAT signaling pathway inhibitor HY-N1447 effectively suppressed the expression of p-JAK1, p-JAK2, and p-STAT3 (Fig. 6C-D).

**Fig. 7:**
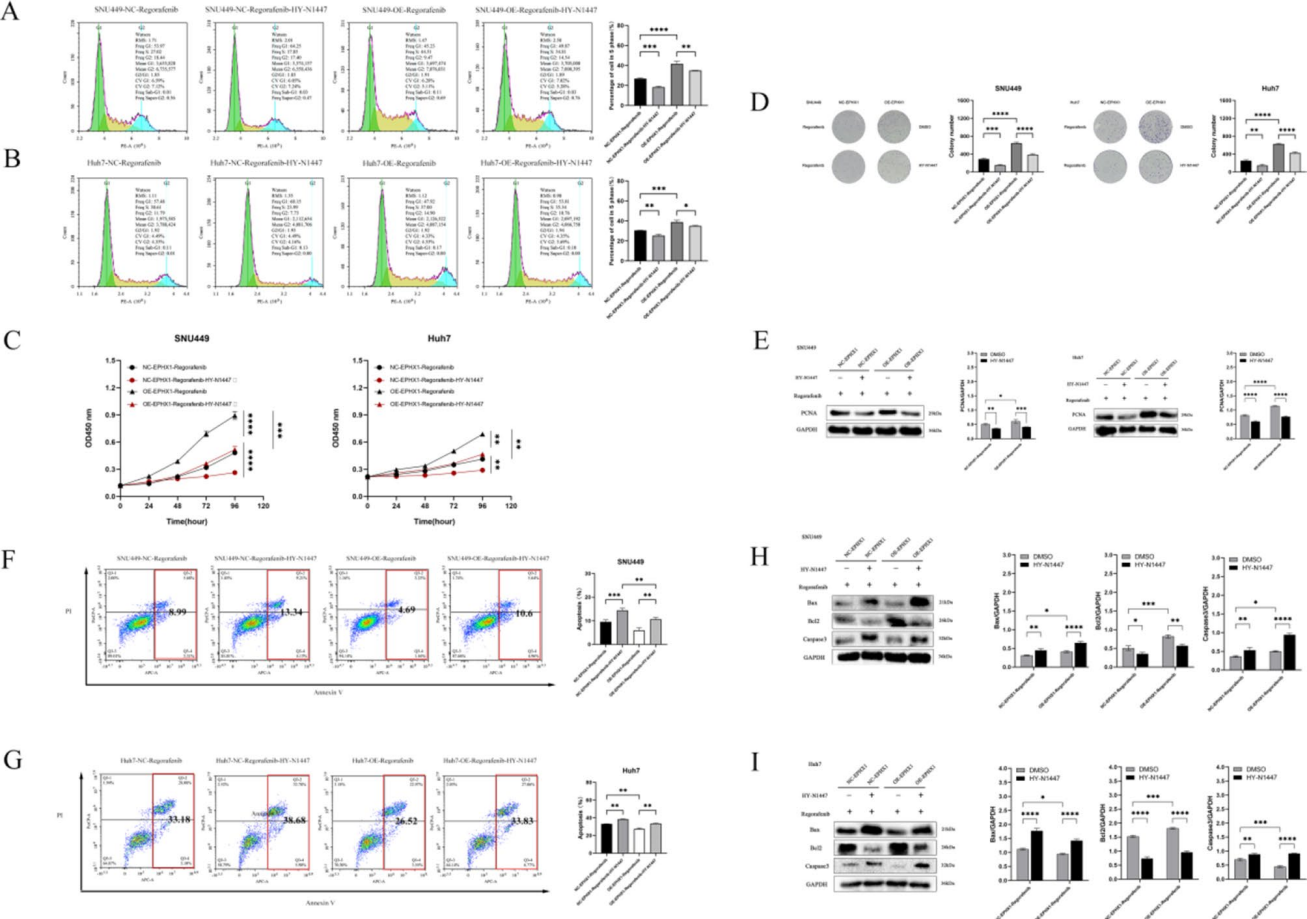
Blockade of the JAK1-JAK2-STAT3 signaling pathway inhibits the promoting effect of EPHX1 on proliferation and suppresses the effect of EPHX1 on apoptosis. (**A**-**B**) Flow cytometry was conducted to perform cell cycle distribution analysis of the cells in NC-EPHX1-Regorafenib ± HY-N1447, OE-EPHX1-Regorafenib ± HY-N1447 groups of SNU449 and Huh7 cells. (**C**) Growth curves measured by CCK-8 showed the growth rates in NC-EPHX1-Regorafenib ± HY-N1447, OE-EPHX1-Regorafenib ± HY-N1447 groups of SNU449 and Huh7 cells. (**D**) Clonogenic assays demonstrated colony formation dynamics in NC-EPHX1-Regorafenib ± HY-N1447 and OE-EPHX1-Regorafenib ± HY-N1447 in SNU449 and Huh7 cell systems. (**E**) Western blot analysis showed proliferating cell nuclear antigen (PCNA) protein expression (normalized to (GAPDH) in NC-EPHX1-Regorafenib ± HY-N1447 and OE-EPHX1-Regorafenib ± HY-N1447groups of SNU449 and Huh7 cells. (**F**-**G**) The amount of apoptosis of NC-EPHX1-Regorafenib ± HY-N1447 and OE-EPHX1-Regorafenib ± HY-N1447 in SNU449 and Huh7 cells were detected by flow cytometry. (**H**-**I**) Western blots were used to detect the expression of Bax, Bcl2 and Caspase-3 in NC-EPHX1-Regorafenib ± HY-N1447 and OE-EPHX1-Regorafenib ± HY-N1447groups of SNU449 and Huh7 cells, * *p* < 0.05, ** *p* < 0.01. *** *p* < 0.001

Pharmacological inhibition of JAK/STAT signaling using HY-N-1447 reversed EPHX1-mediated resistance: Combined treatment with HY-N-1447 and regorafenib reduced S-phase cell populations (Fig. 7A-B), suppressed proliferation (CCK-8: Fig. 7C; colony formation: Fig. 7D), downregulated PCNA expression (Fig. 7E), and increased apoptosis (Fig. 7F-J). Mechanistically, HY-N-1447 restored regorafenib-induced apoptosis markers (upregulated Bax/caspase-3, downregulated Bcl-2) regardless of EPHX1 status (Fig. 7H-I).

## Conclusion

Through genetic manipulation of EPHX1 combined with regorafenib treatment and JAK/STAT pathway inhibition, we demonstrate that: (1) Regorafenib exerts antitumor effects by suppressing JAK1/JAK2/STAT3 signaling, thereby inhibiting proliferation and inducing apoptosis; (2) EPHX1 drives regorafenib resistance via constitutive activation of JAK/STAT signaling, which counteracts the drug’s therapeutic effects. These findings establish EPHX1 as a critical mediator of chemoresistance in HCC and highlight JAK/STAT pathway modulation as a promising therapeutic strategy.

## Discussion

Despite the continuous development of new chemotherapeutic agents, drug resistance remains a major obstacle in cancer treatment, often leading to tumor recurrence after remission [22]. This study systematically investigates the role and molecular mechanism of EPHX1 in hepatocellular carcinoma (HCC) cell viability, apoptosis, and regorafenib resistance. EPHX1 is localized to the 1q42.1 region of chromosome 1, containing 9 exons and 8 introns [12]. There are two key polymorphisms in its coding region: Tyr113His in exon 3 (reducing enzyme activity by 40–50%) and His139Arg in exon 4 (increasing enzyme activity by 25%) [23]. These polymorphisms are associated with various diseases such as HCC [24], lung cancer [14], and colorectal cancer [16]. Notably, the association between Tyr113His and HCC risk remains controversial and requires further validation [25].

As a biotransformation enzyme, EPHX1 can catalyze the hydrolysis of epoxides into low-activity, highly water-soluble products. It also enhances the carcinogenicity of benzo[a]pyrene through interaction with hepatitis B virus (HBV) cleavage proteins [26] and participates in drug metabolism, which is associated with 5-fluorouracil resistance [21] and carbamazepine metabolism [27]. Importantly, its polymorphisms (e.g., His139Arg) can affect drug efficacy by regulating metabolite hydrolysis [28]. However, this study did not explore its impact on regorafenib resistance, which is a limitation. As an approved second-line tyrosine kinase inhibitor (TKI) for HCC [29], regorafenib exerts its effects by inhibiting kinases such as RAF and VEGFR [30] and is the only therapy with survival benefits after sorafenib progression [31]. This study is the first to find that EPHX1 is highly expressed in HCC tissues. Its overexpression can increase the IC50 of regorafenib, weaken the drug’s inhibition on apoptosis and proliferation, while knockdown enhances drug efficacy (validated by both in vitro and in vivo experiments), suggesting that EPHX1 promotes regorafenib resistance.

Transcriptome analysis showed significant changes in the JAK/STAT pathway after EPHX1 knockdown. This pathway is a conserved signaling cascade activated by cytokines, regulating proliferation, apoptosis, and other processes, and plays a crucial role in HCC progression [32, 33]. Regorafenib can function by inhibiting STAT3 phosphorylation [34]. Mechanistically, EPHX1 is an interferon (IFNs)-dependent transcript, and its expression is upregulated by IFN signaling [35]. IFNs are classic activators of JAK/STAT: upon binding to receptors, they activate JAK1/2/Tyk2, phosphorylate STAT1/2, and the latter forms dimers that translocate into the nucleus to regulate target genes [36]. EPHX1 can affect IFN production by regulating cellular redox status [37], while IFN-induced upregulation of EPHX1 can enhance the IFN pathway through antioxidant activity [38], forming an “EPHX1-IFN-JAK/STAT” regulatory axis, which reasonably explains the indirect activation of JAK/STAT by EPHX1. In HCC, abnormal IFN signaling can lead to JAK/STAT dysregulation. In this study, EPHX1 overexpression was associated with JAK/STAT activation, and the specific interaction mechanism needs further exploration.

In conclusion, EPHX1 inhibits the anti-tumor effect of regorafenib by activating the JAK/STAT pathway, and the JAK/STAT inhibitor HY-N-1447 can reverse the resistance induced by EPHX1, suggesting that EPHX1 targeting JAK/STAT is an important mechanism of regorafenib resistance in HCC. Additionally, EPHX1 may participate in drug resistance by regulating apoptosis-related proteins. Future studies should clarify the direct interaction between EPHX1 and IFN signaling components, as well as the impact of EPHX1 polymorphisms on regorafenib response, to provide new targets for overcoming drug resistance.

## Supplementary Information

Below is the link to the electronic supplementary material.

Supplementary Material 1

Supplementary Material 2: Supplementary Figure 1: Induction of Regorafenib resistance by EPHX1 in hepatocarcinoma cell lines. (A) The IC50 values of Regorafenib were detected by CCK-8 assays in four HCC cell lines. (B) Western blot analysis showed the levels of EPHX1 in four HCC cell lines after Regorafenib treatment. * p < 0.05，** p < 0.01，*** p < 0.001.

Supplementary Material 3: Supplementary Figure 2: Induction of Regorafenib resistance by EPHX1 in hepatocarcinoma cell lines. (A) Transfection efficiency and mRNA expression level and protein expression level of EPHX1 of knock-down in SNU449. (B) Knock-down in Huh7. (C) Overexpression in SNU449. (D) Overexpression in Huh7.

## Data Availability

No datasets were generated or analysed during the current study.

## Abbreviations

HCC: Hepatocellular carcinoma
EPHX1: microsomal epoxide hydrolase 1
TIMER: Tumor Immune Estimation Resource
FDA: Food and Drug Administration
IC50: Half maximal inhibitory concentration
DGE-seq: Digital Gene Expression Profiling
IHC: Immunohistochemistry
DMEM: Dulbecco’s Modified Eagle Medium
FBS: fetal bovine serum
PBS: Phosphate Buffered Saline
RT-qPCR: Reverse transcription-quantitative PCR
PFS: progression-free survival
OS: overall survival
PCNA: Proliferating Cell Nuclear Antigen
BCL-2: B-cell lymphoma-2
BAX: B-cell lymphoma-2-Associated X
Caspase3: cysteinyl aspartate specific proteinase
JAK1: janus kinase 1
p-JAK1: Phosphorylation janus kinase 1
JAK2: janus kinase 2
p-JAK2: Phosphorylation janus kinase 2
STAT3: signal transducer and activator of transcription 3
p-STAT3: Phosphorylation signal transducer and activator of transcription 3
