# Supplementary material for: EPHX1 enhances drug resistance to regorafenib by activating the JAK/STAT signaling pathway in hepatocellular carcinoma cell lines
Source: Hereditas. 2025 Jul 31;162:148. doi: 10.1186/s41065-025-00517-1 (PMC12315303; doi:10.1186/s41065-025-00517-1)
Supplement: Supplementary file 1 — Supplementary Material 1 [file 41065_2025_517_MOESM1_ESM.pdf]

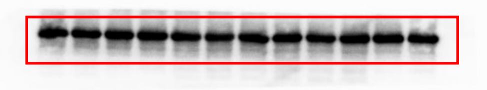

Fig 1 C the Gapdh of 1-6 Normal and Tumor

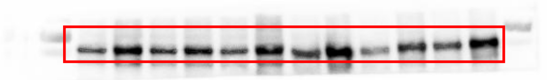

Fig 1 C the EPHX1 of 1-6 Normal and Tumor

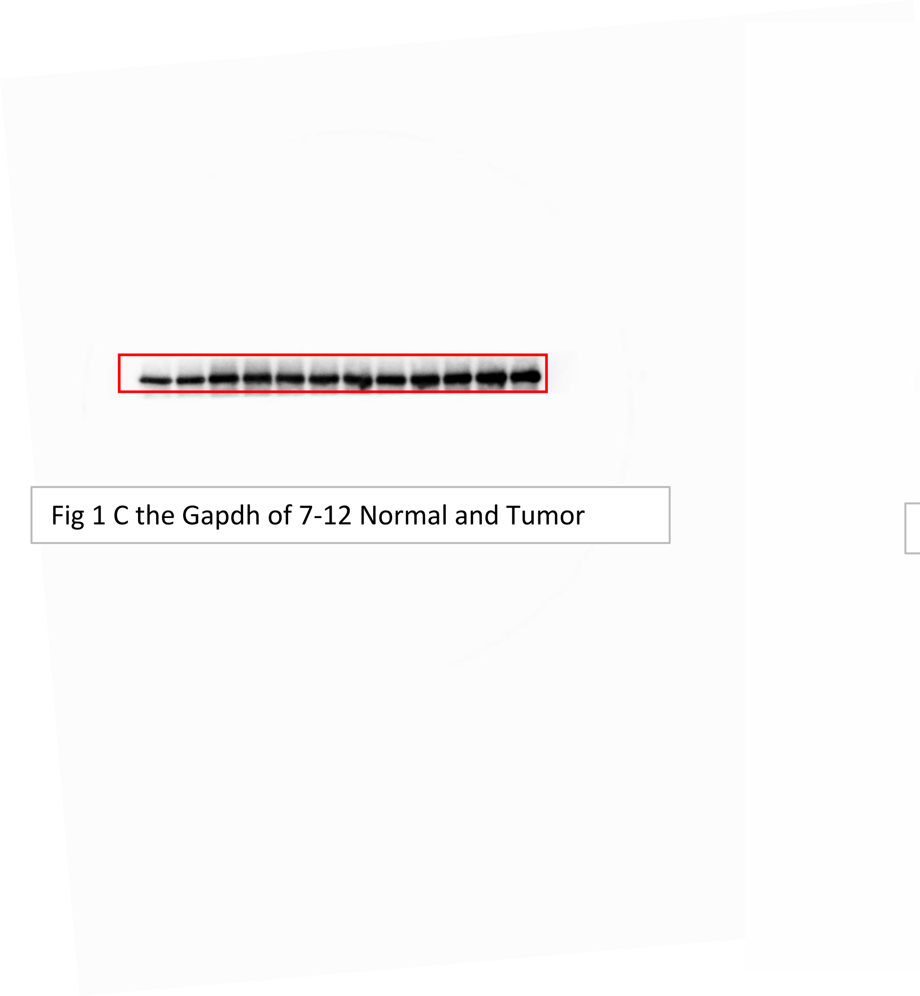

Fig 1 C the Gapdh of 7-12 Normal and Tumor

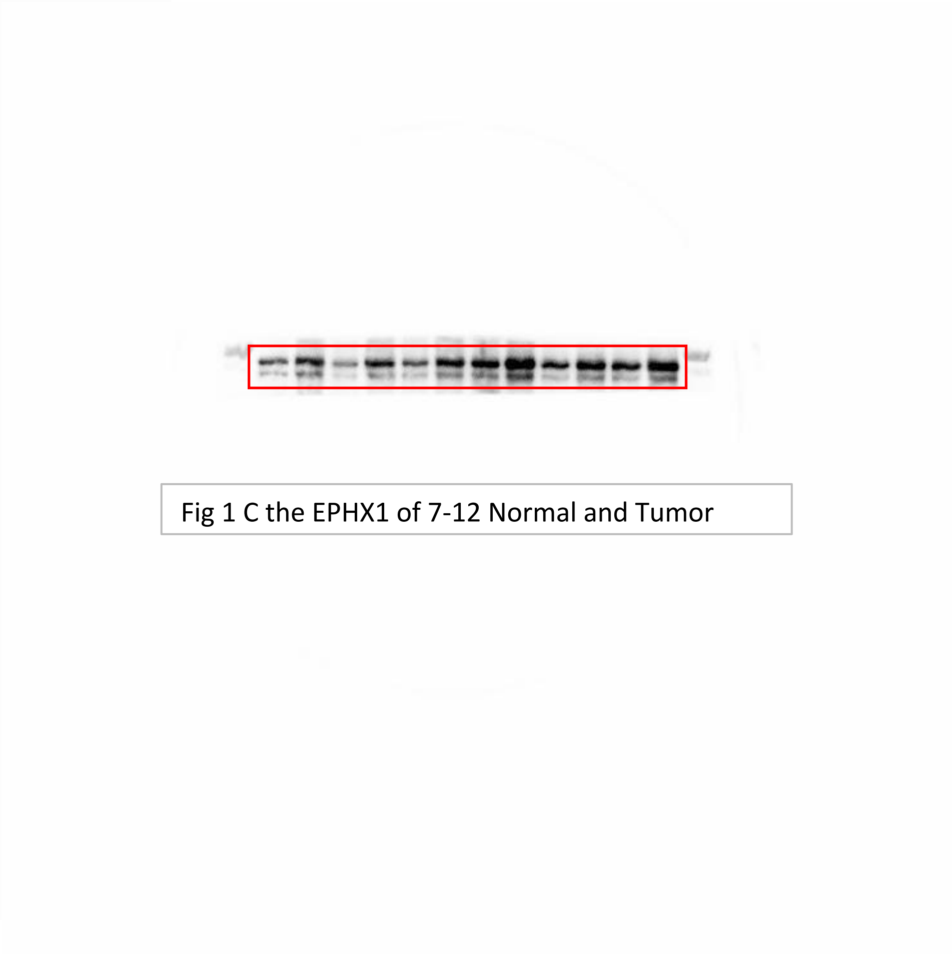

Fig 1 C the EPHX1 of 7-12 Normal and Tumor

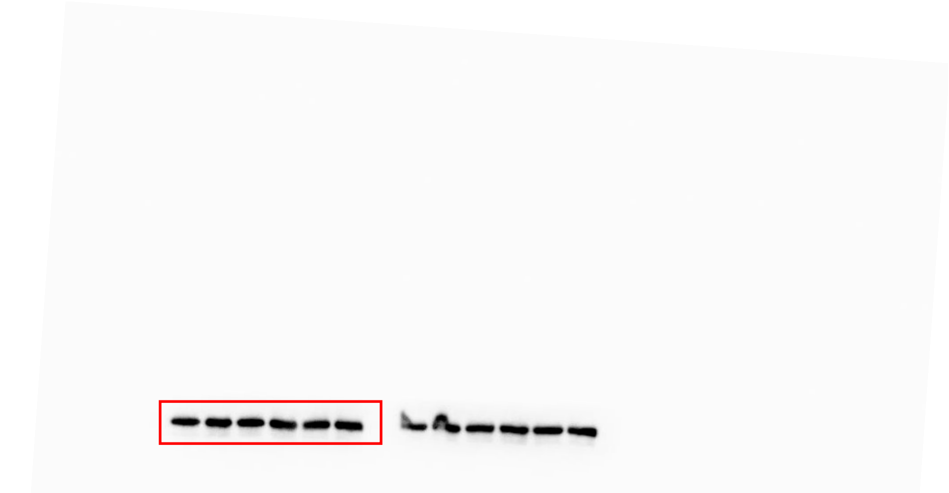

Fig 1 C the Gapdh of 13-15 Normal and Tumor

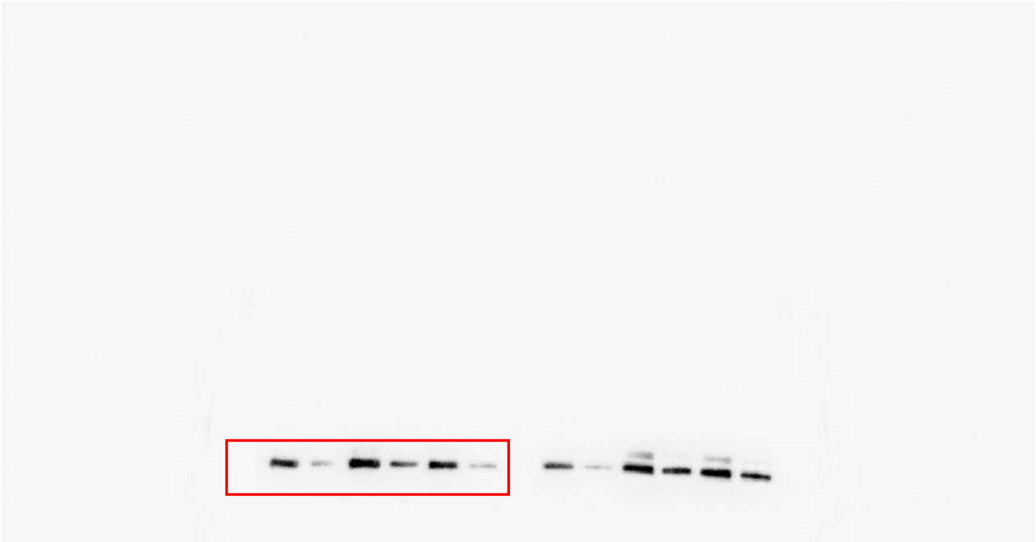

Fig 1 C the EPHX1 of 13-15 Normal and Tumor

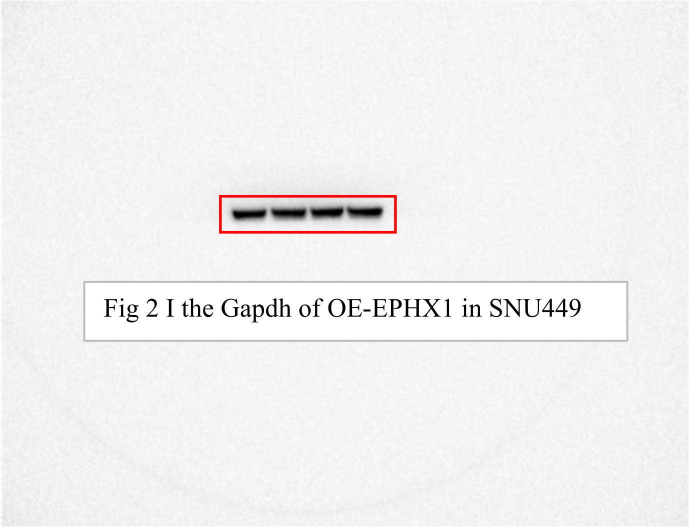

Fig 2 I the Gapdh of OE-EPHX1 in SNU449

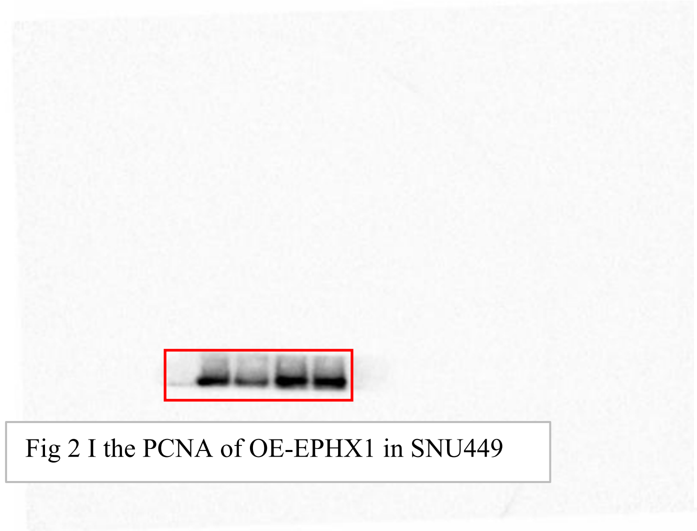

Fig 2 I the PCNA of OE-EPHX1 in SNU449

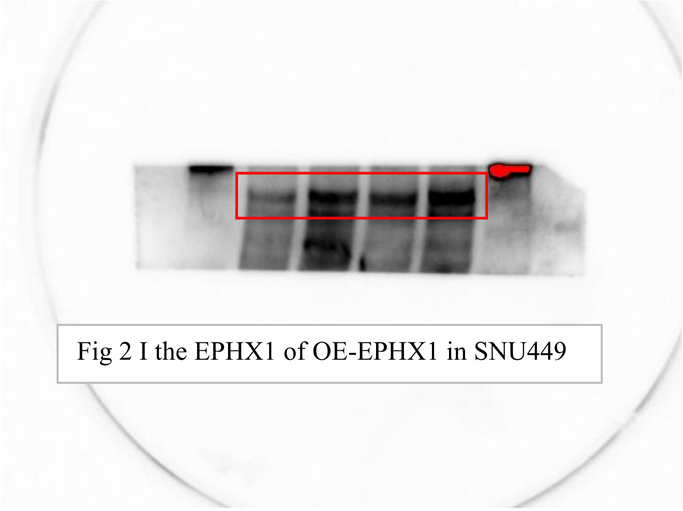

Fig 2 I the EPHX1 of OE-EPHX1 in SNU449

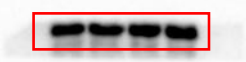

Fig 2 I the Gapdh of OE-EPHX1 in Huh7

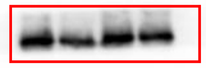

Fig 2 I the PCNA of OE-EPHX1 in Huh7

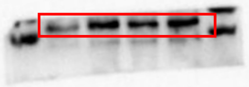

Fig 2 I the EPHX1 of OE-EPHX1 in Huh7

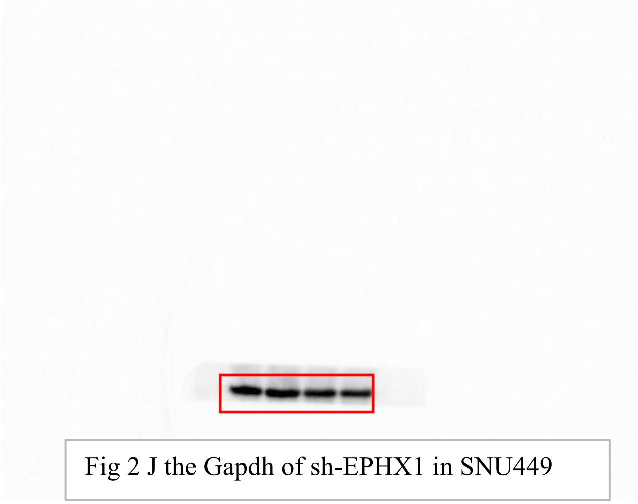
Fig 2 J the Gapdh of sh-EPHX1 in SNU449

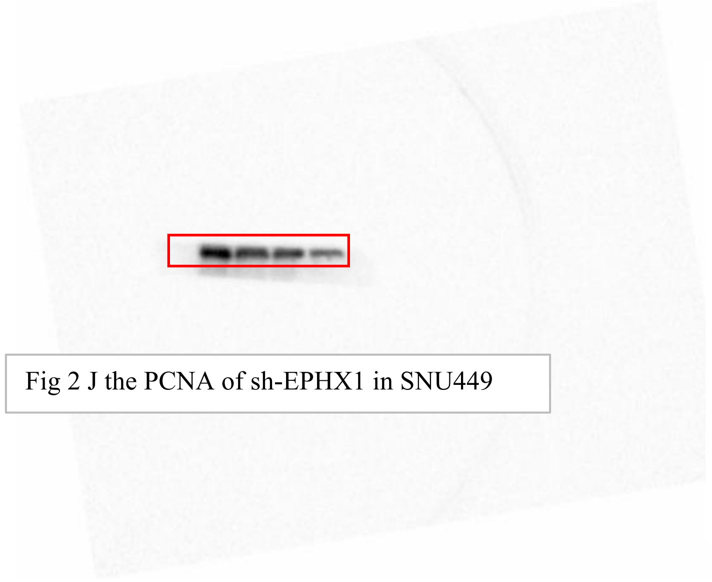
Fig 2 J the PCNA of sh-EPHX1 in SNU449

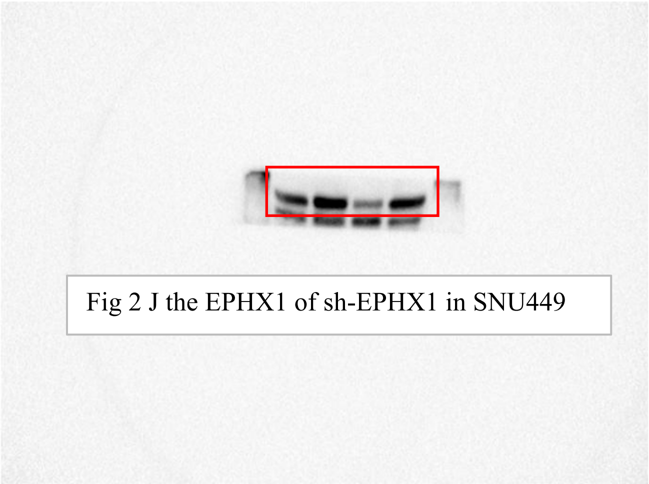
Fig 2 J the EPHX1 of sh-EPHX1 in SNU449

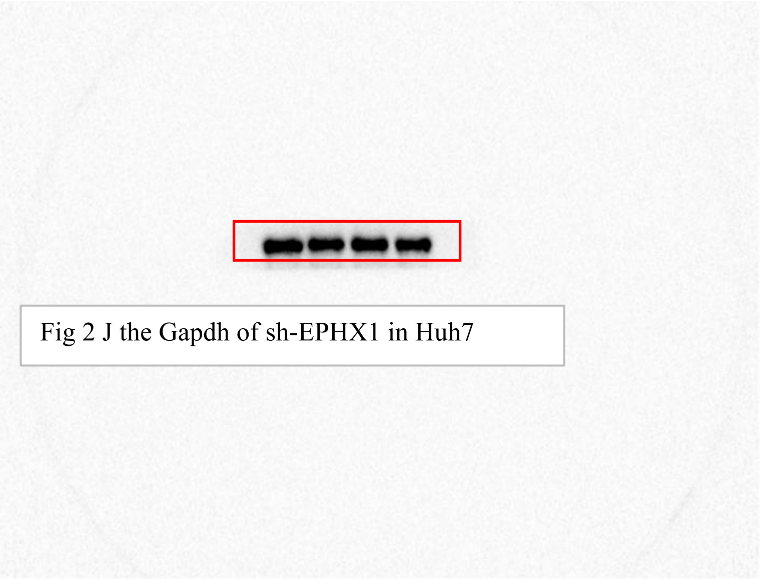

Fig 2 J the Gapdh of sh-EPHX1 in Huh7

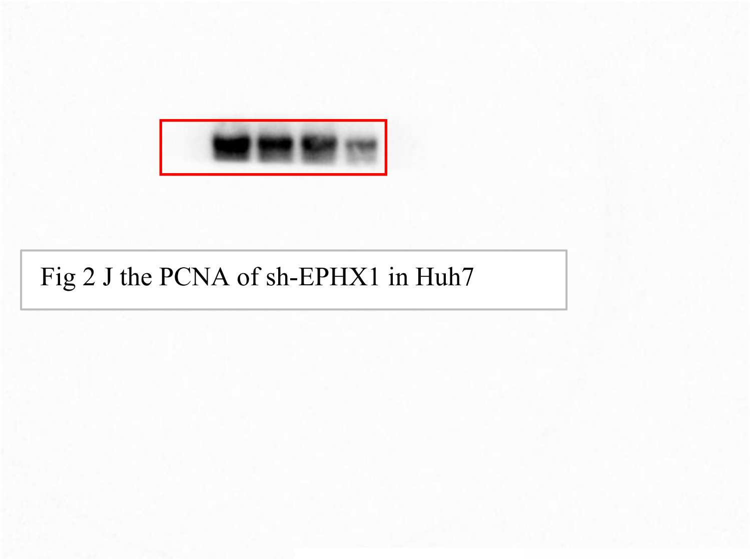

Fig 2 J the PCNA of sh-EPHX1 in Huh7

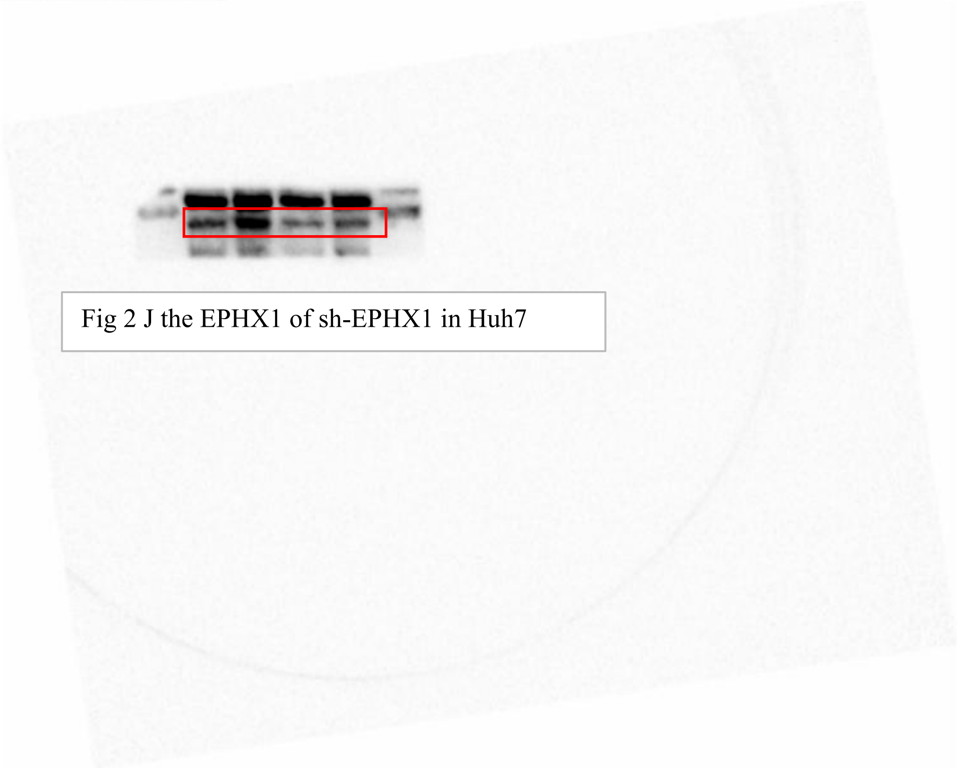

Fig 2 J the EPHX1 of sh-EPHX1 in Huh7

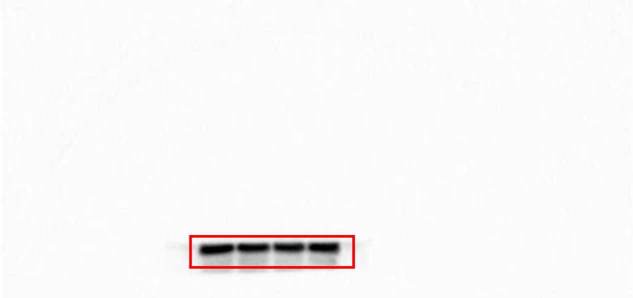

Fig 3 E the Gapdh of OE-EPHX1 in SNU449

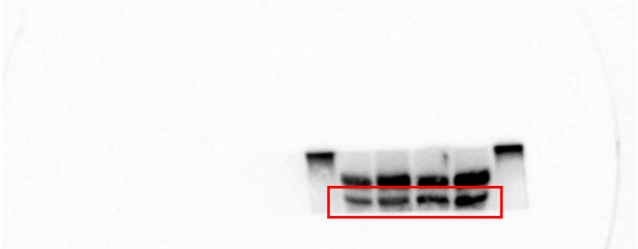

Fig 3 E the EPHX1 of OE-EPHX1 in SNU449

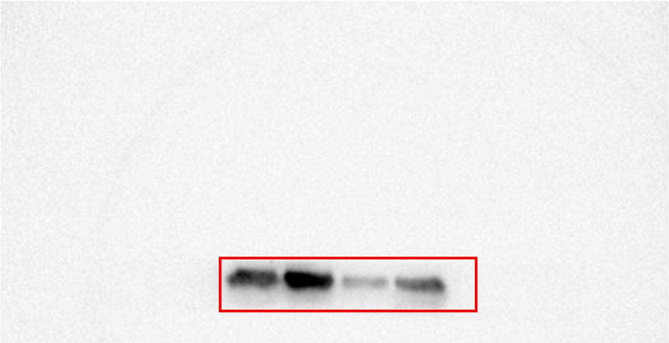

Fig 3 E the Bax of OE-EPHX1 in SNU449

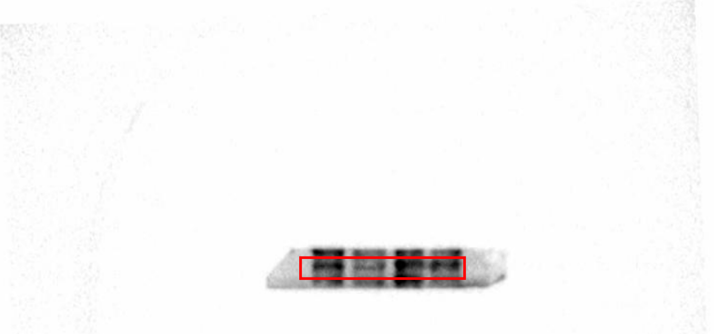

Fig 3 E the Bcl2 of OE-EPHX1 in SNU449

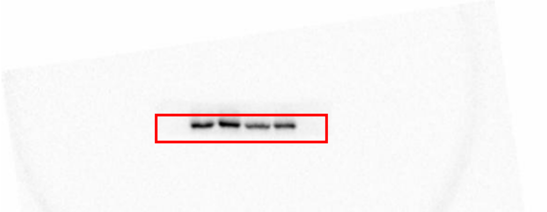

Fig 3 E the Caspase3 of OE-EPHX1 in SNU449

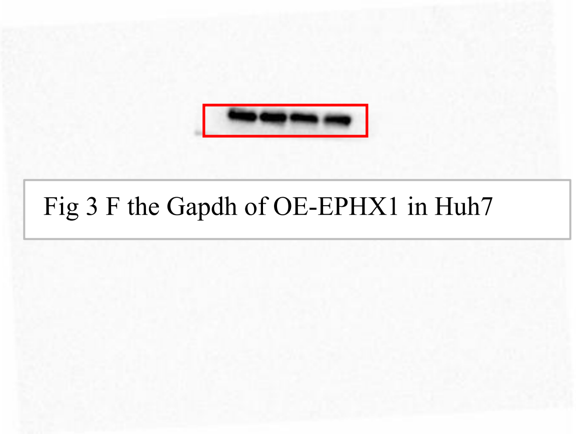

Fig 3 F the Gapdh of OE-EPHX1 in Huh7

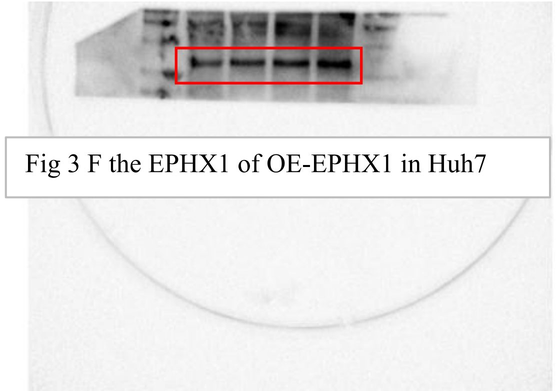

Fig 3 F the EPHX1 of OE-EPHX1 in Huh7

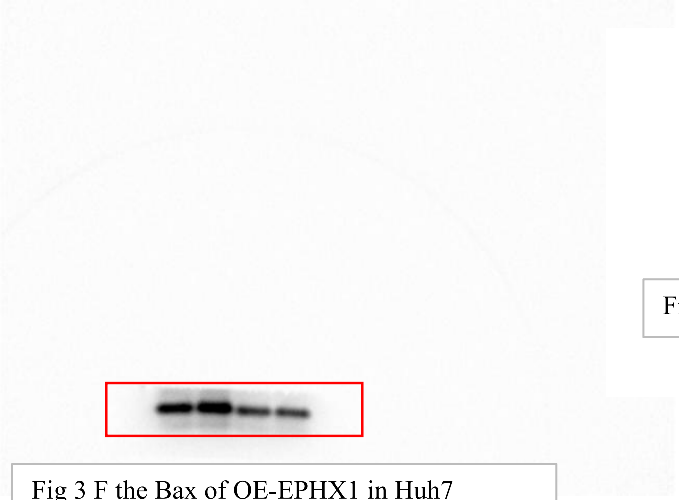

Fig 3 F the Bax of OE-EPHX1 in Huh7

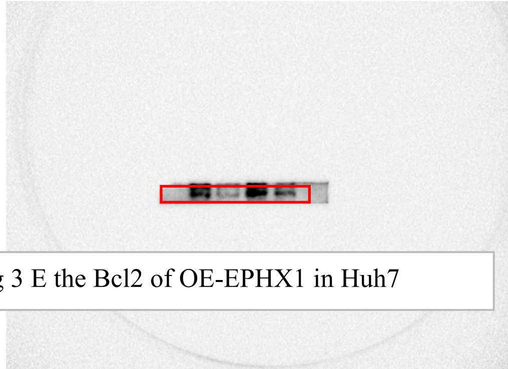

Fig 3 E the Bcl2 of OE-EPHX1 in Huh7

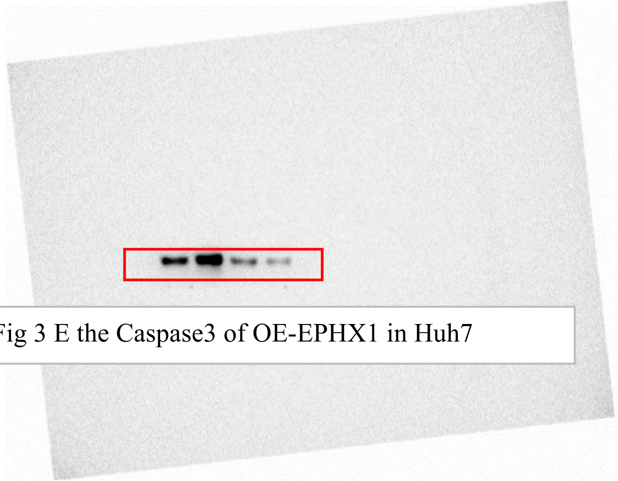

Fig 3 E the Caspase3 of OE-EPHX1 in Huh7

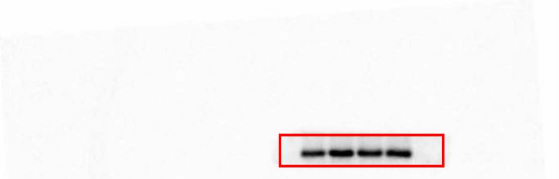

Fig 3 G the Gapdh of sh-EPHX1 in SNU449

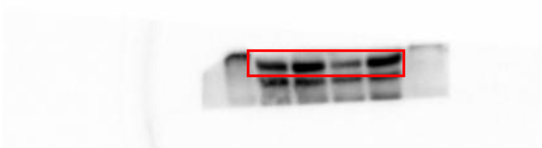

Fig 3 G the EPHX1 of sh-EPHX1 in SNU449

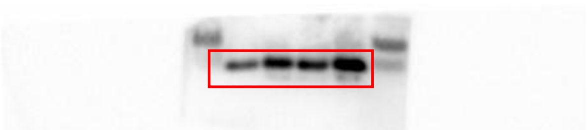

Fig 3 G the Bax of sh-EPHX1 in SNU449

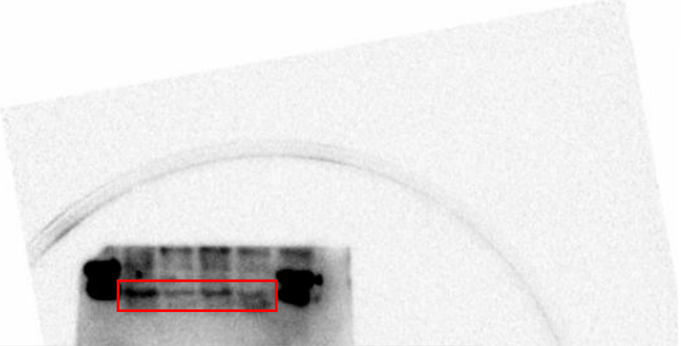

Fig 3 G the Bcl2 of sh-EPHX1 in SNU449

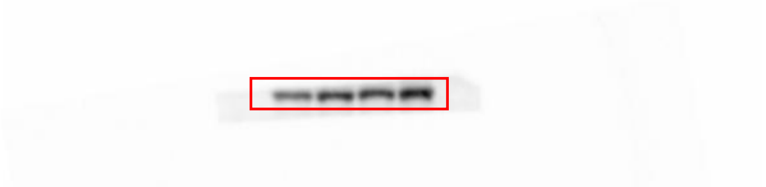

Fig 3 G the Caspase3 of sh-EPHX1 in SNU449

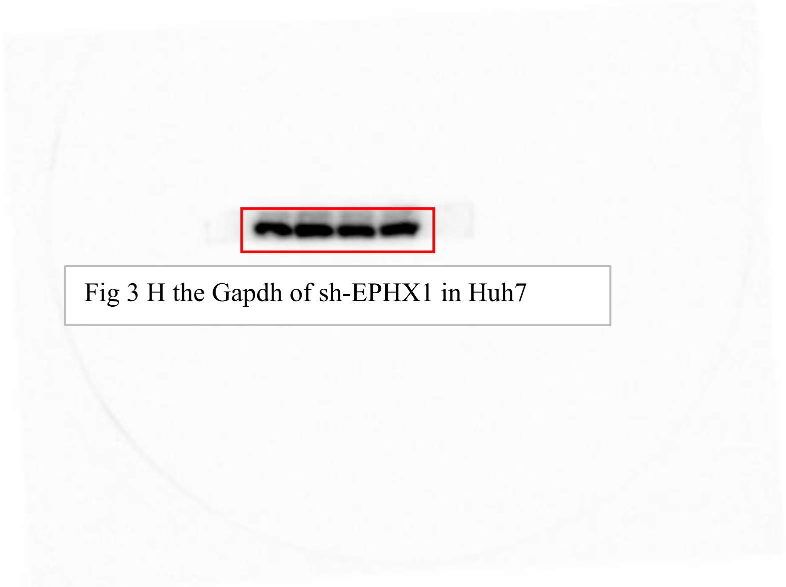

Fig 3 H the Gapdh of sh-EPHX1 in Huh7

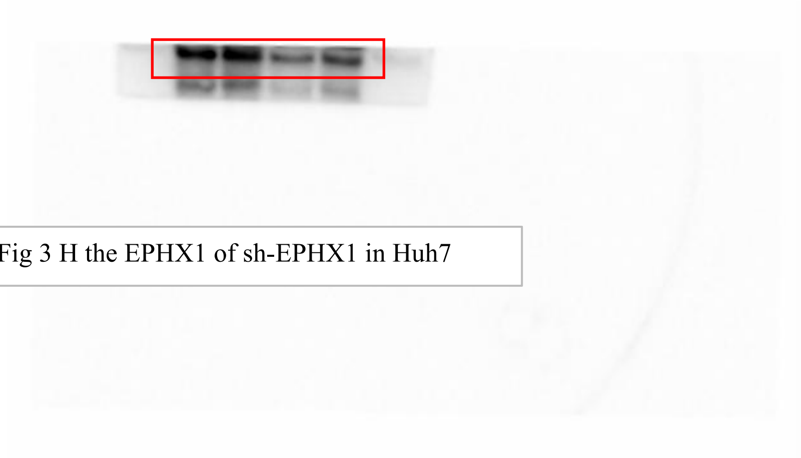

Fig 3 H the EPHX1 of sh-EPHX1 in Huh7

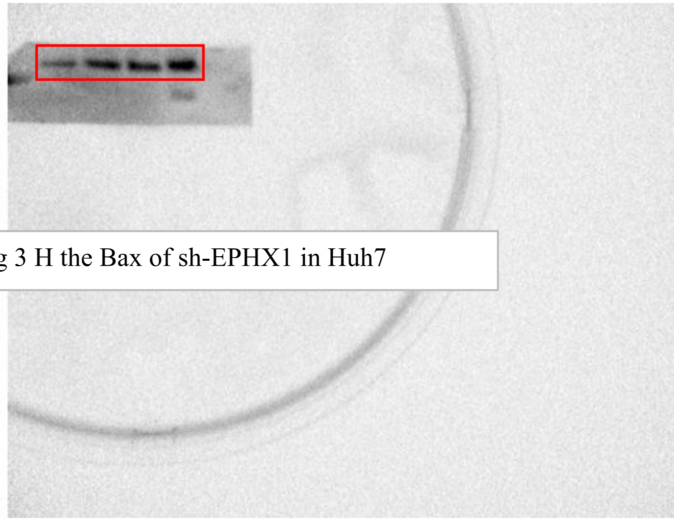

Fig 3 H the Bax of sh-EPHX1 in Huh7

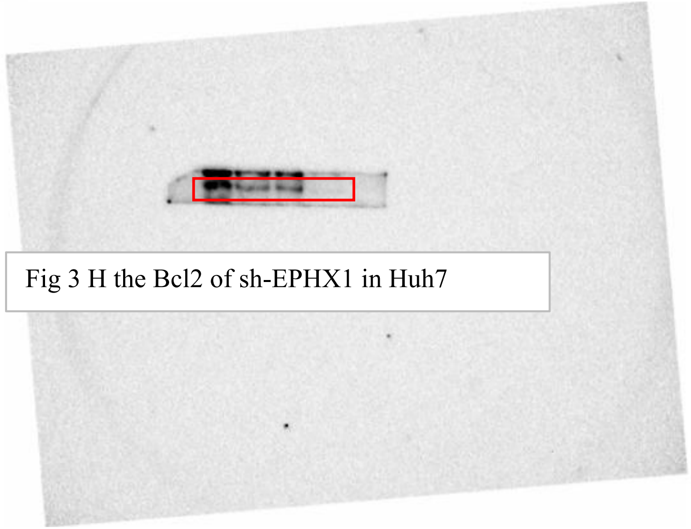

Fig 3 H the Bcl2 of sh-EPHX1 in Huh7

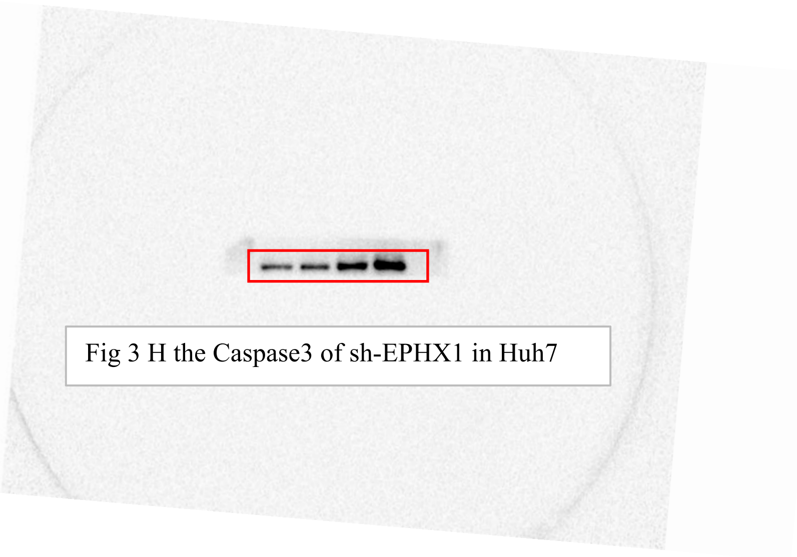

Fig 3 H the Caspase3 of sh-EPHX1 in Huh7

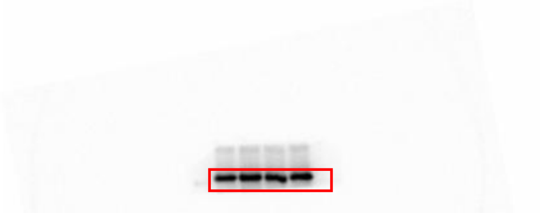
Figure 6 A the Gapdh of OE-EPHX1 in SNU449

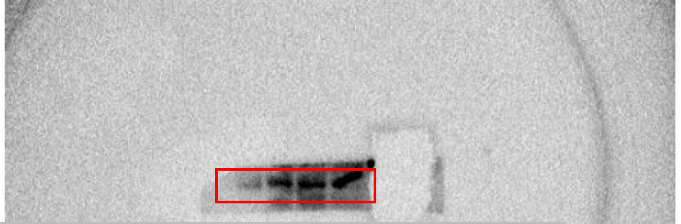
Figure 6 A the EPHX1 of OE-EPHX1 in SNU449

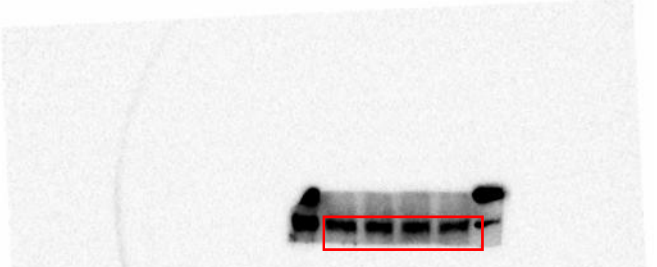
Figure 6 A the JAK1 of OE-EPHX1 in SNU449

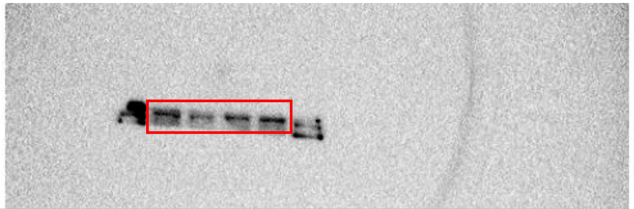
Figure 6 A the p-JAK1 of OE-EPHX1 in SNU449

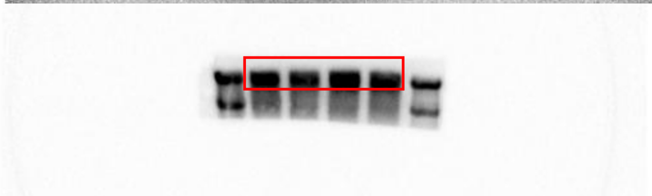
Figure 6 A the JAK2 of OE-EPHX1 in SNU449

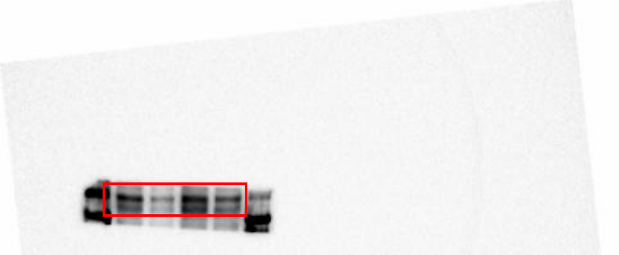
Figure 6 A the p-JAK2 of OE-EPHX1 in SNU449

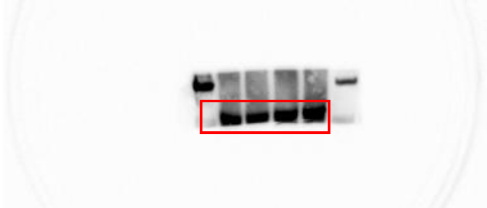
Figure 6 A the STAT3 of OE-EPHX1 in SNU449

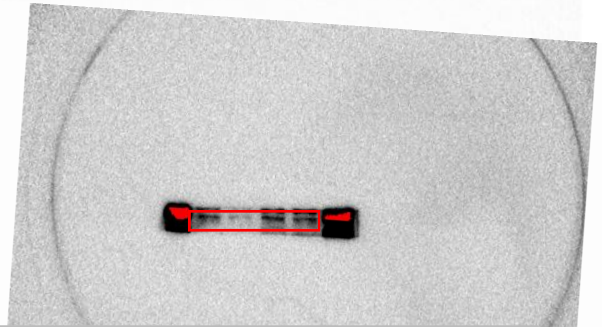
Figure 6 A the p-STAT3 of OE-EPHX1 in SNU449

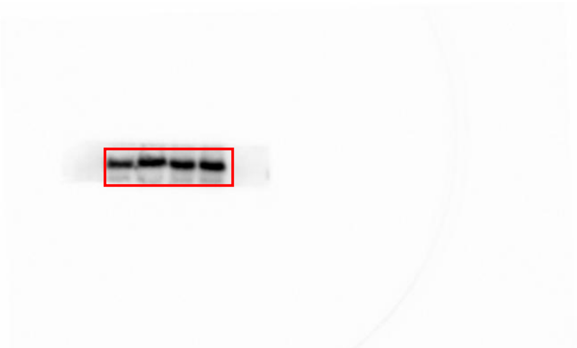
Figure 6 A the Gapdh of sh-EPHX1 in SNU449

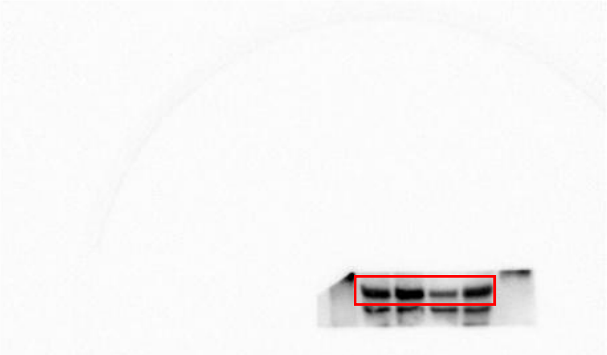
Figure 6 A the EPHX1 of sh-EPHX1 in SNU449

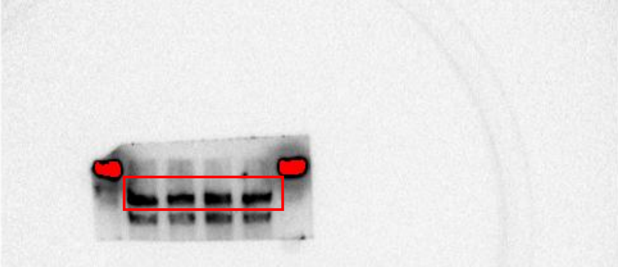
Figure 6 A the JAK1 of sh-EPHX1 in SNU449

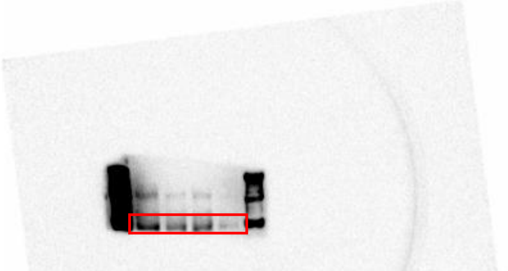
Figure 6 A the p-JAK1 of sh-EPHX1 in SNU449

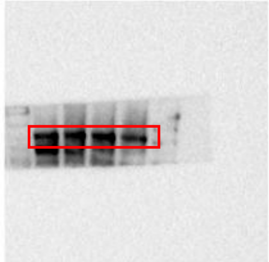
Figure 6 A the JAK2 of sh-EPHX1 in SNU449

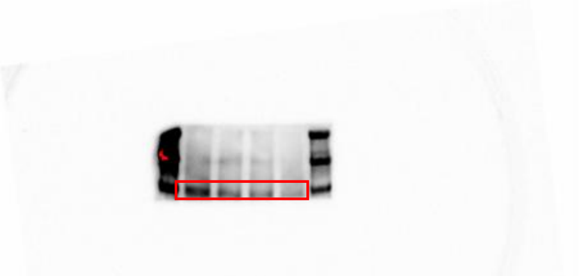
Figure 6 A the p-JAK2 of sh-EPHX1 in SNU449

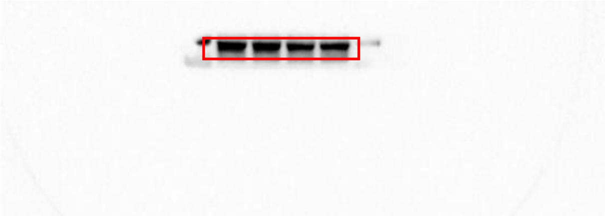
Figure 6 A the STAT3 of sh-EPHX1 in SNU449

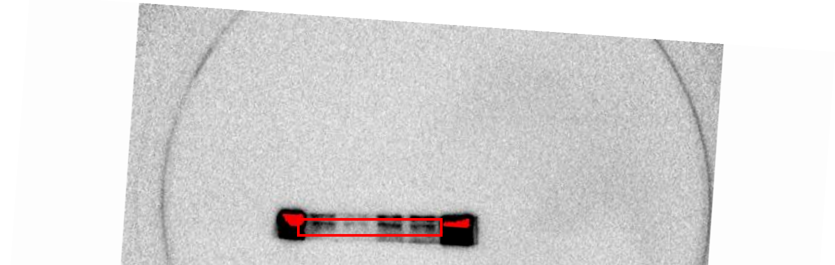
Figure 6 A the p-STAT3 of sh-EPHX1 in SNU449

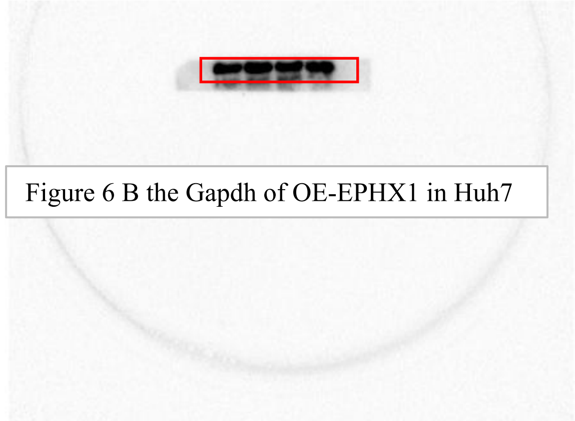

Figure 6 B the Gapdh of OE-EPHX1 in Huh7

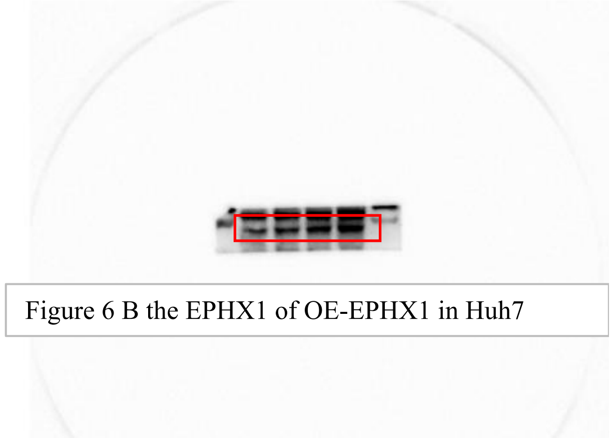

Figure 6 B the EPHX1 of OE-EPHX1 in Huh7

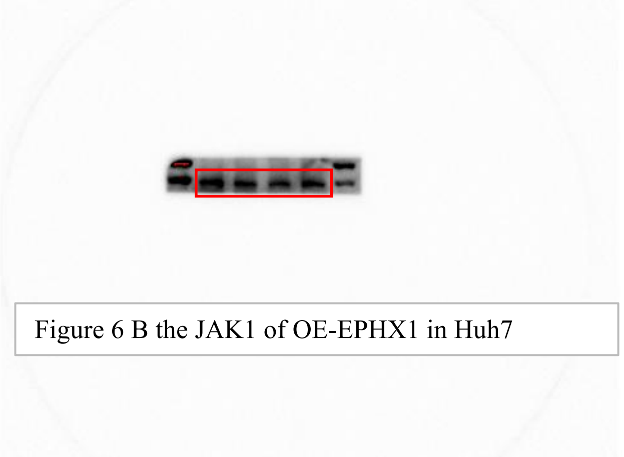

Figure 6 B the JAK1 of OE-EPHX1 in Huh7

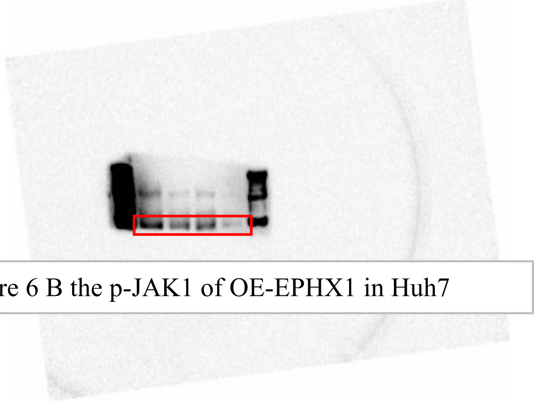

Figure 6 B the p-JAK1 of OE-EPHX1 in Huh7

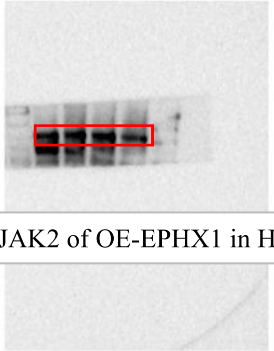

Figure 6 B the JAK2 of OE-EPHX1 in Huh7

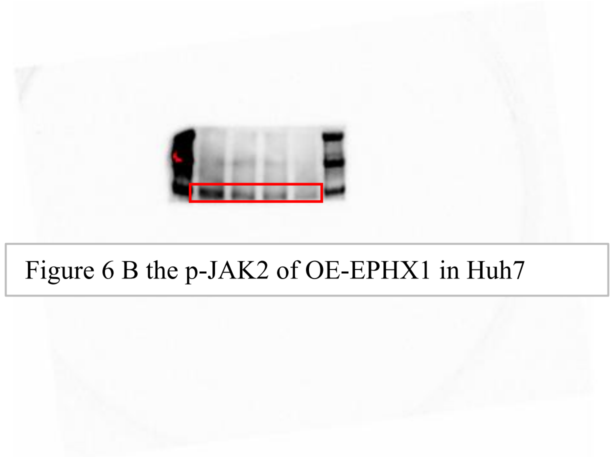

Figure 6 B the p-JAK2 of OE-EPHX1 in Huh7

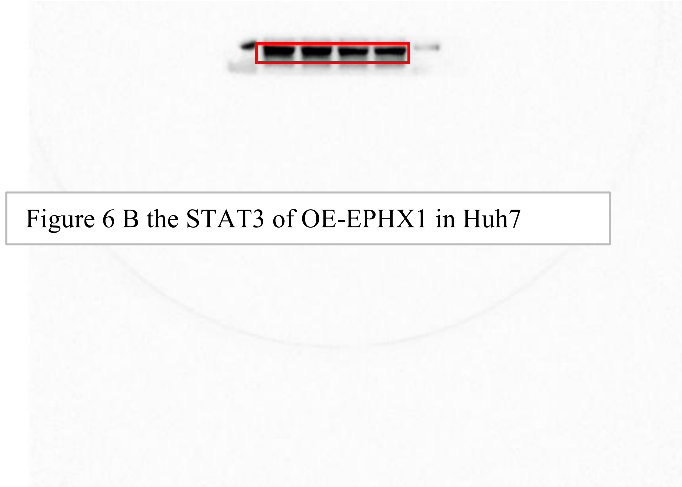

Figure 6 B the STAT3 of OE-EPHX1 in Huh7

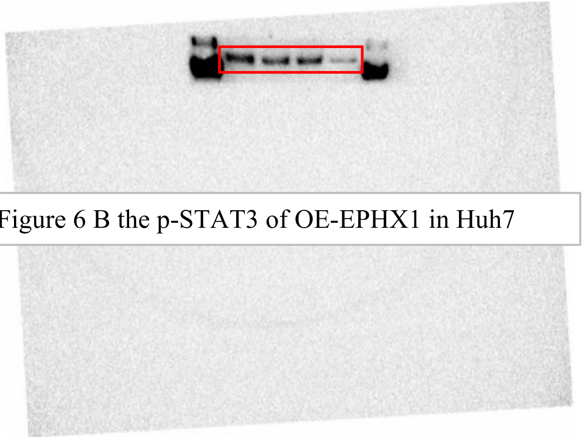

Figure 6 B the p-STAT3 of OE-EPHX1 in Huh7

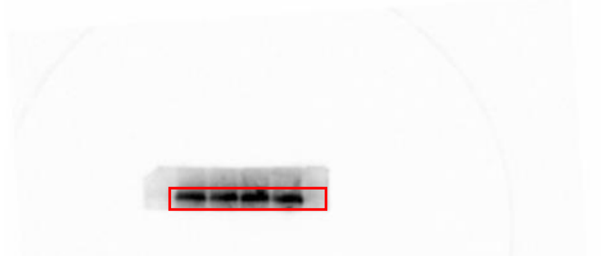

Figure 6 B the Gapdh of sh-EPHX1 in Huh7

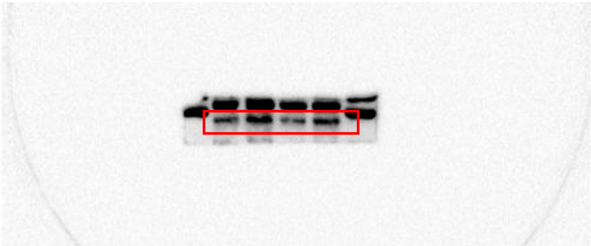

Figure 6 B the EPHX1 of sh-EPHX1 in Huh7

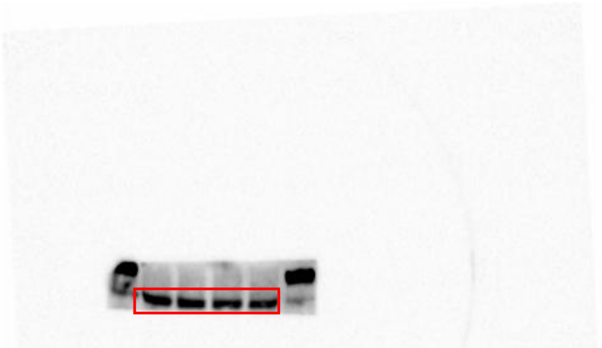

Figure 6 B the JAK1 of sh-EPHX1 in Huh7

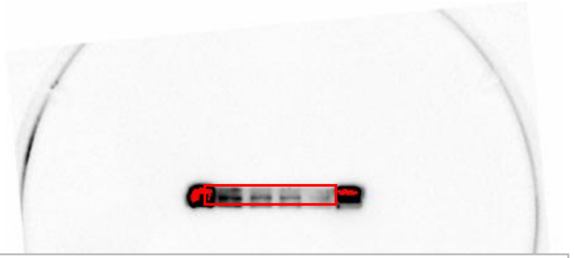

Figure 6 B the p-JAK1 of sh-EPHX1 in Huh7

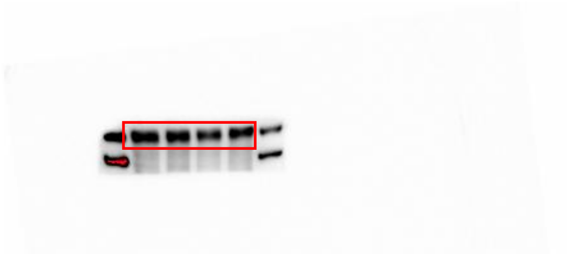

Figure 6 B the JAK2 of sh-EPHX1 in Huh7

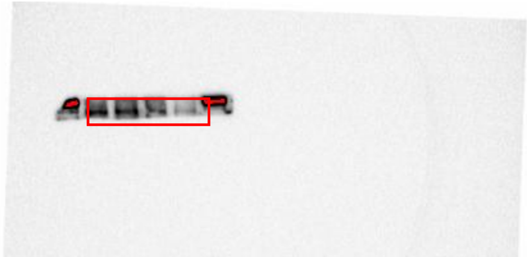

Figure 6 B the p-JAK2 of OE-EPHX1 in Huh7

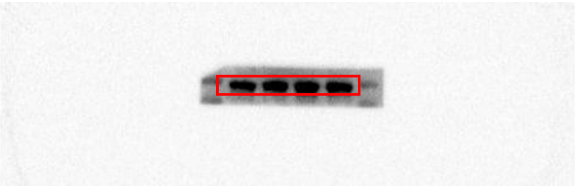

Figure 6 B the STAT3 of OE-EPHX1 in Huh7

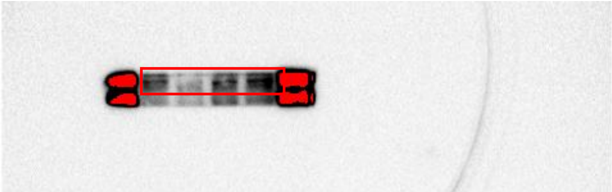

Figure 6 B the p-STAT3 of OE-EPHX1 in Huh7

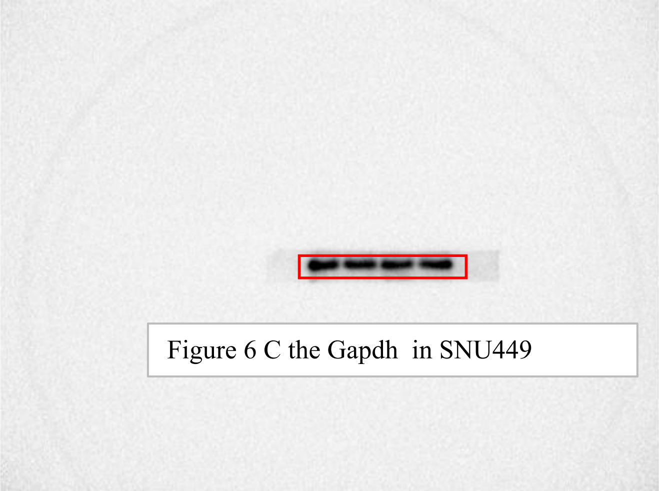
Figure 6 C the Gapdh in SNU449

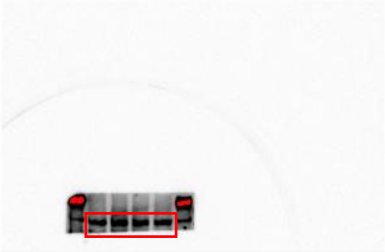
Figure 6 C the JAK1 in SNU449

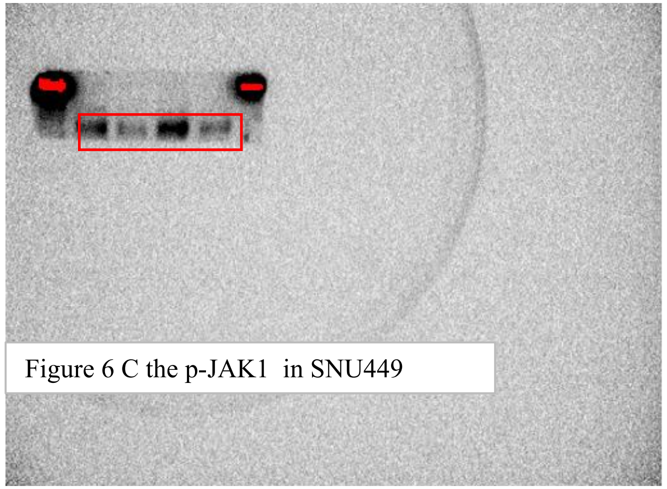
Figure 6 C the p-JAK1 in SNU449

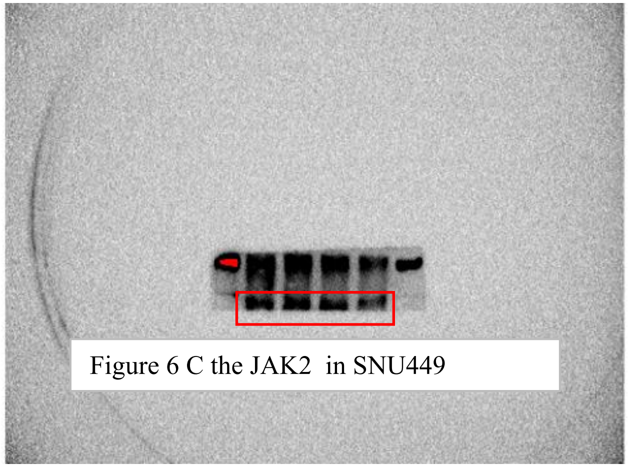
Figure 6 C the JAK2 in SNU449

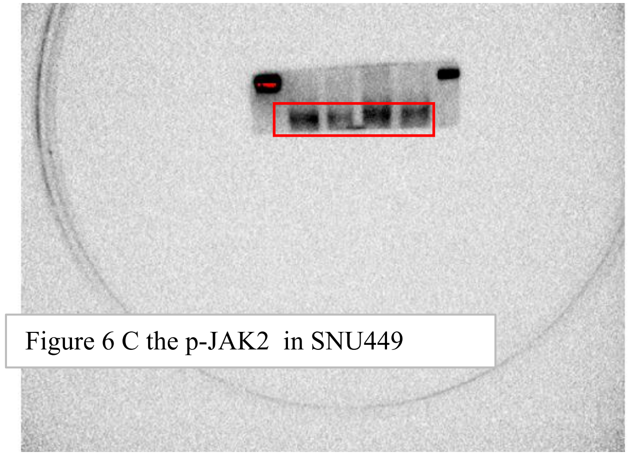
Figure 6 C the p-JAK2 in SNU449

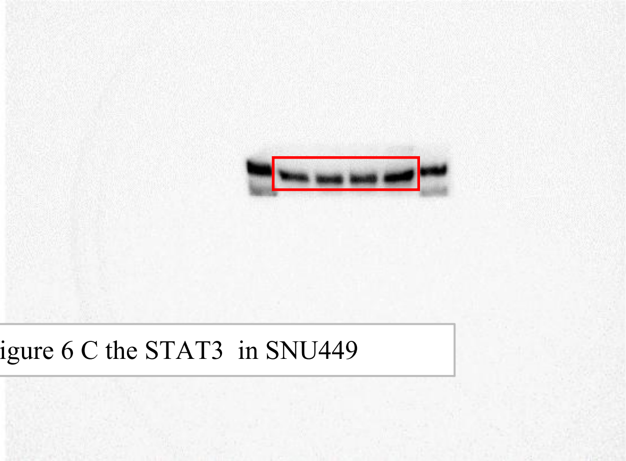
Figure 6 C the STAT3 in SNU449

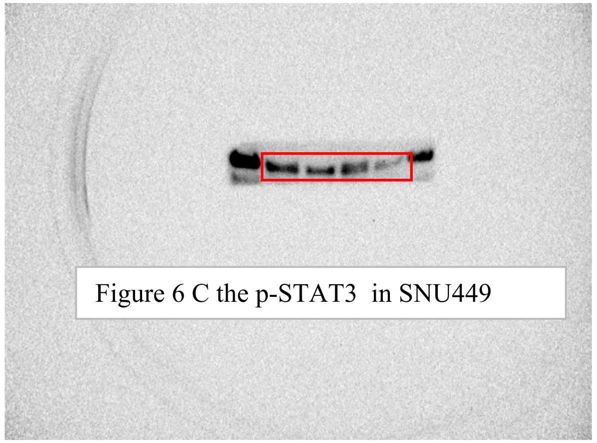
Figure 6 C the p-STAT3 in SNU449

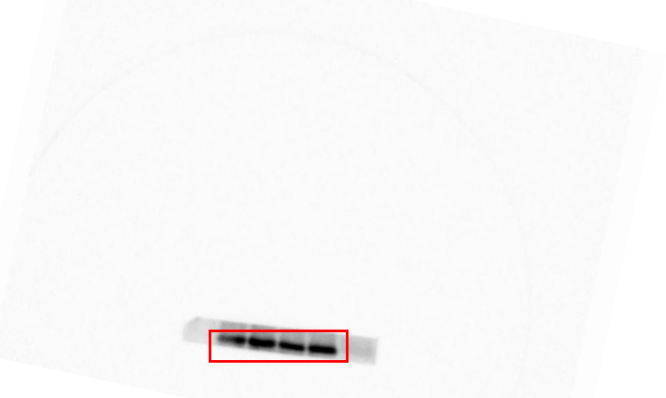
Figure 6 D the Gapdh in Huh7

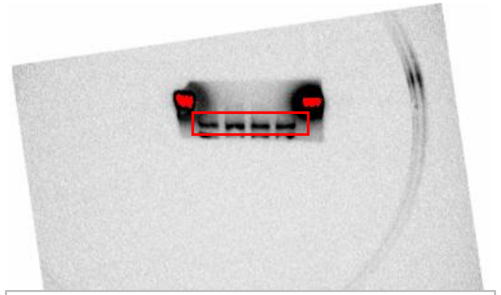
Figure 6 D the JAK1 in Huh7

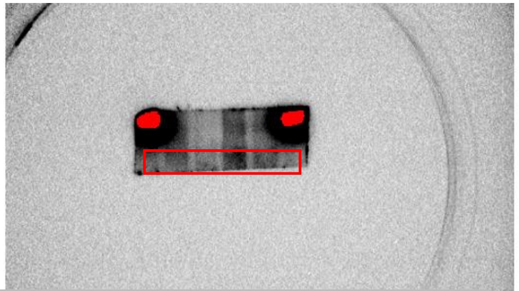
Figure 6 D the p-JAK1 in Huh7

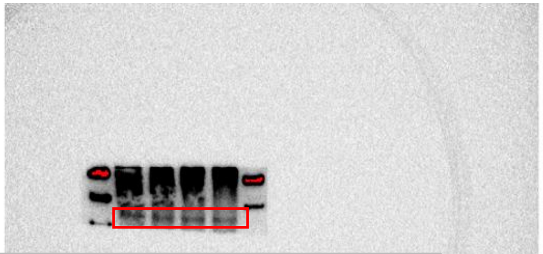
Figure 6 D the JAK2 in Huh7

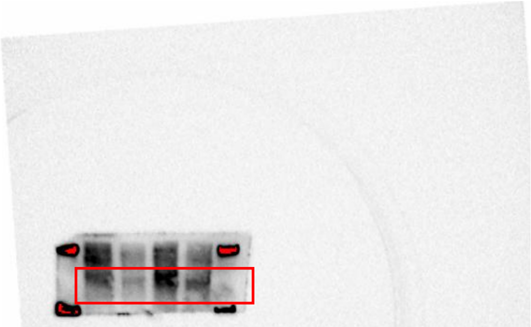
Figure 6 D the p-JAK2 in Huh7

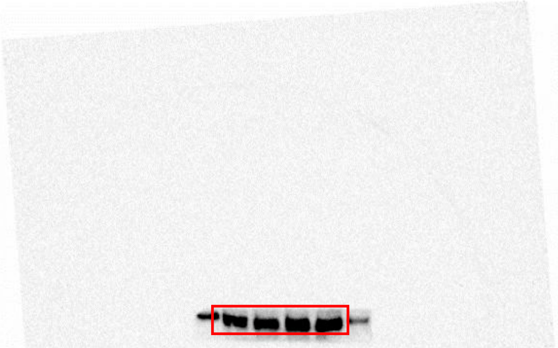
Figure 6 D the STAT3 in Huh7

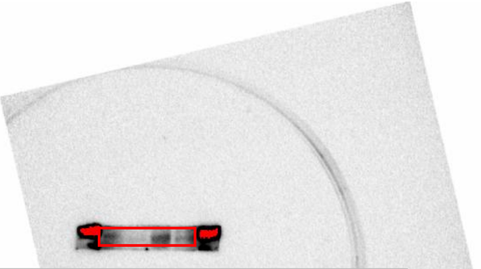
Figure 6 D the p-STAT3 in Huh7

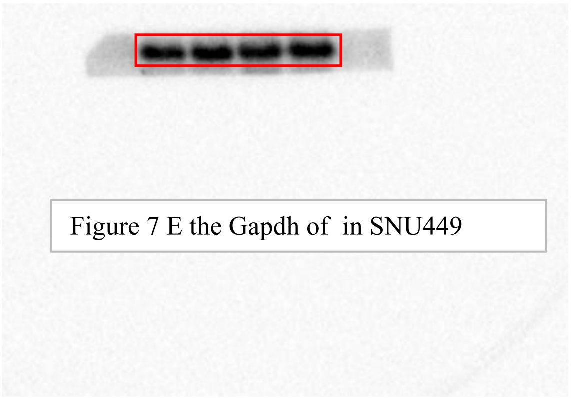
Figure 7 E the Gapdh of in SNU449

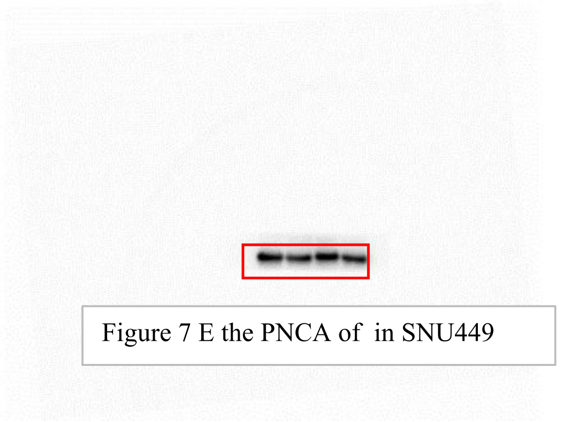
Figure 7 E the PNCA of in SNU449

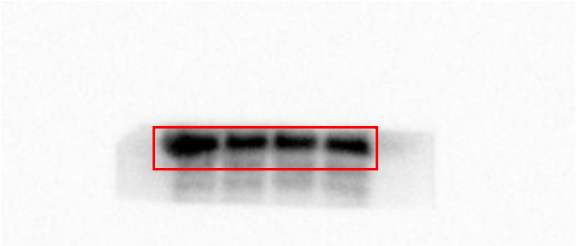

Figure 7 E the Gapdh of in Huh7

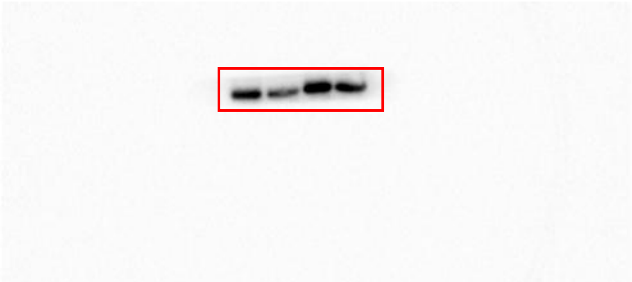

Figure 7 E the PNCA of in Huh7

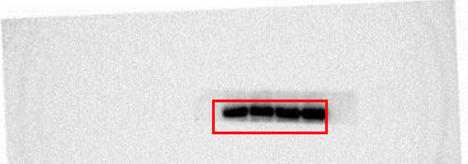

Figure 7 H the Gapdh of in SNU449

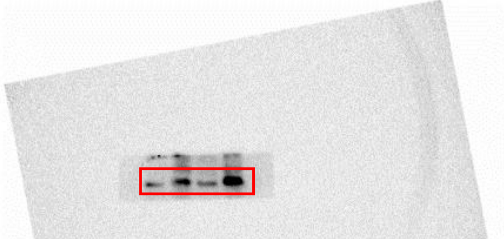

Figure 7 H the Bax of in SNU449

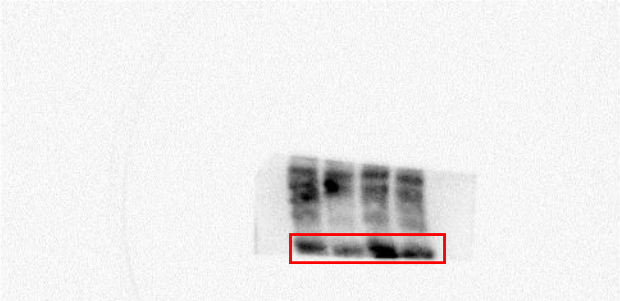

Figure 7 H the Bcl2 of in SNU449

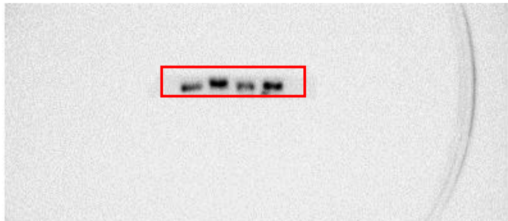

Figure 7 H theCaspase3 of in SNU449

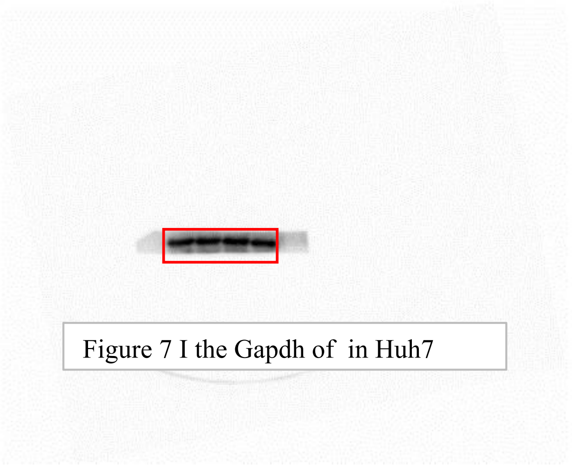

Figure 7 I the Gapdh of in Huh7

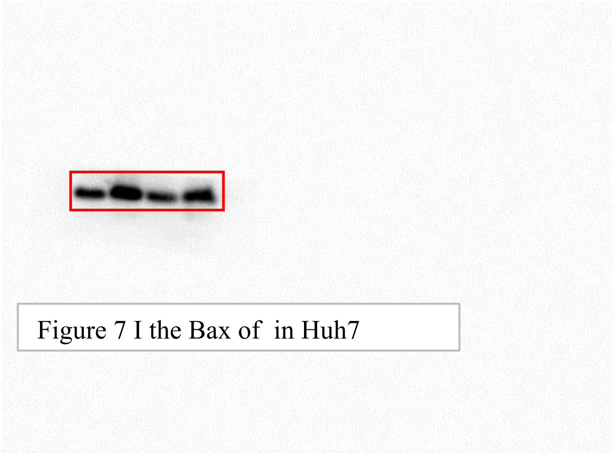

Figure 7 I the Bax of in Huh7

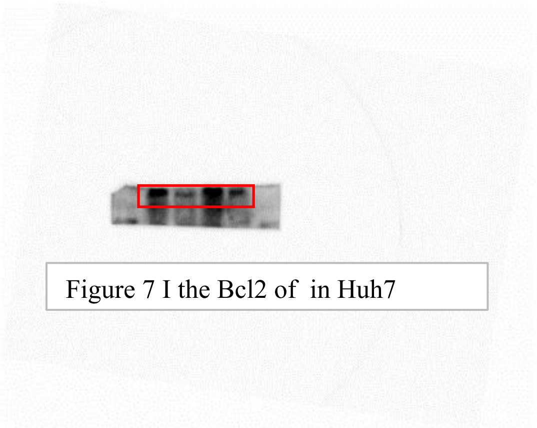

Figure 7 I the Bcl2 of in Huh7

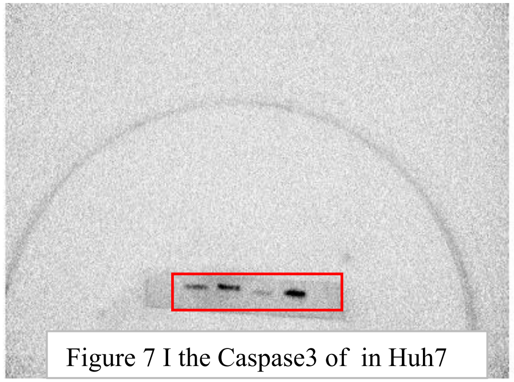

Figure 7 I the Caspase3 of in Huh7

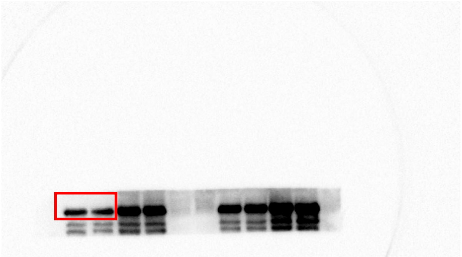

Supplementary Figure 1 B the Gapdh of in SNU449

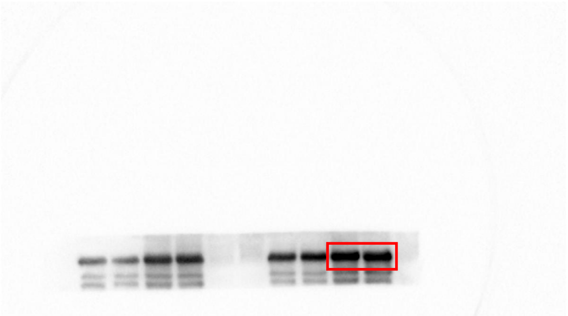

Supplementary Figure 1 B the Gapdh of in Huh7

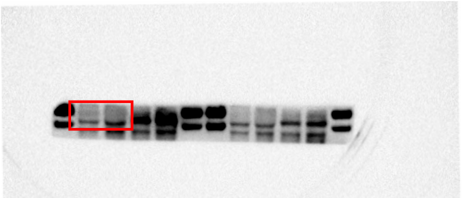

Supplementary Figure 1 B the EPHX1 of in SNU449

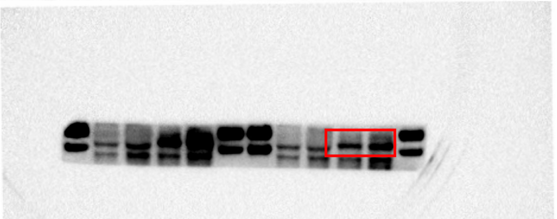

Supplementary Figure 1 B the EPHX1 of in Huh7

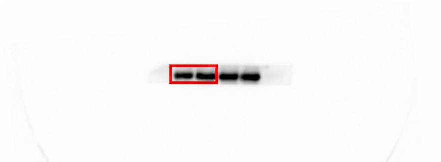

Supplementary Figure 1 B the Gapdh of in MHCC-97H

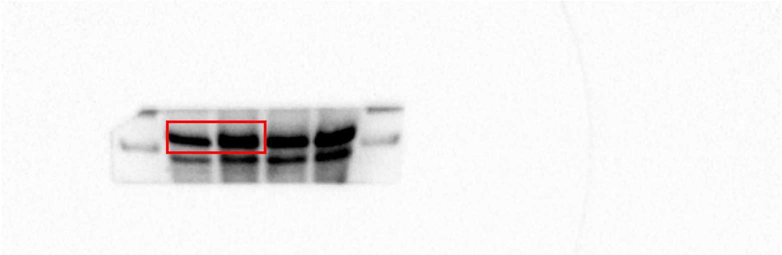

Supplementary Figure 1 B the EPHX1 of in MHCC-97H

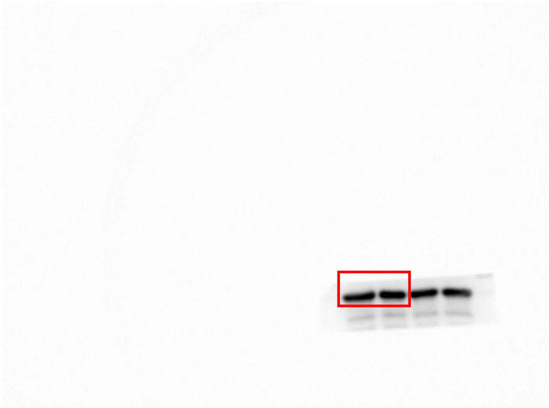

Supplementary Figure 1 B the Gapdh of in SNU387

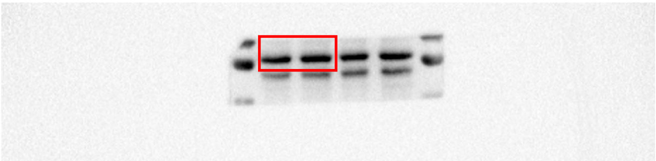

Supplementary Figure 1 B the EPHX1 of in SNU387

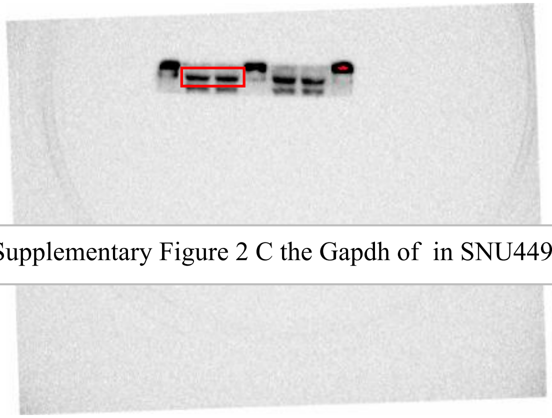

Supplementary Figure 2 C the Gapdh of in SNU449

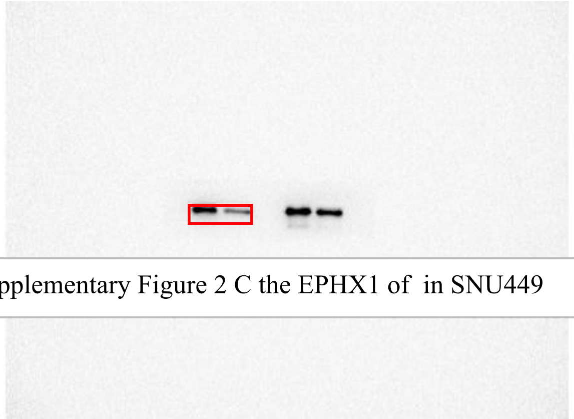

Supplementary Figure 2 C the EPHX1 of in SNU449

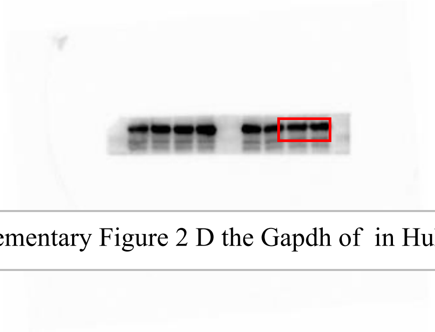

Supplementary Figure 2 D the Gapdh of in Huh7

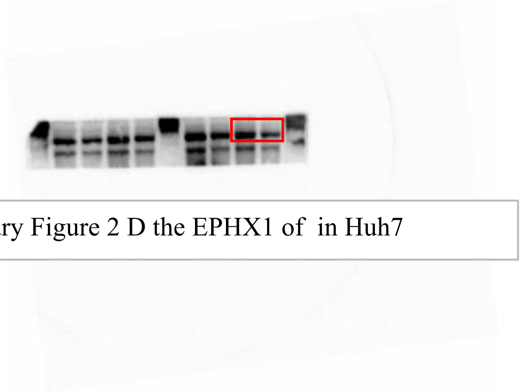

Supplementary Figure 2 D the EPHX1 of in Huh7
