# Supplementary material for: EPHX1 enhances drug resistance to regorafenib by activating the JAK/STAT signaling pathway in hepatocellular carcinoma cell lines
Source: Hereditas. 2025 Jul 31;162:148. doi: 10.1186/s41065-025-00517-1 (PMC12315303; doi:10.1186/s41065-025-00517-1)
Supplement: Supplementary file 2 — Supplementary Material 2: Supplementary Figure 1: Induction of Regorafenib resistance by EPHX1 in hepatocarcinoma cell lines. (A) The IC50 values of Regorafenib were detected by CCK-8 assays in four HCC cell lines. (B) Western blot analysis showed the levels of EPHX1 in four HCC cell lines after Regorafenib treatment. * p < 0.05，** p < 0.01，*** p < 0.001. [file 41065_2025_517_MOESM2_ESM.docx]

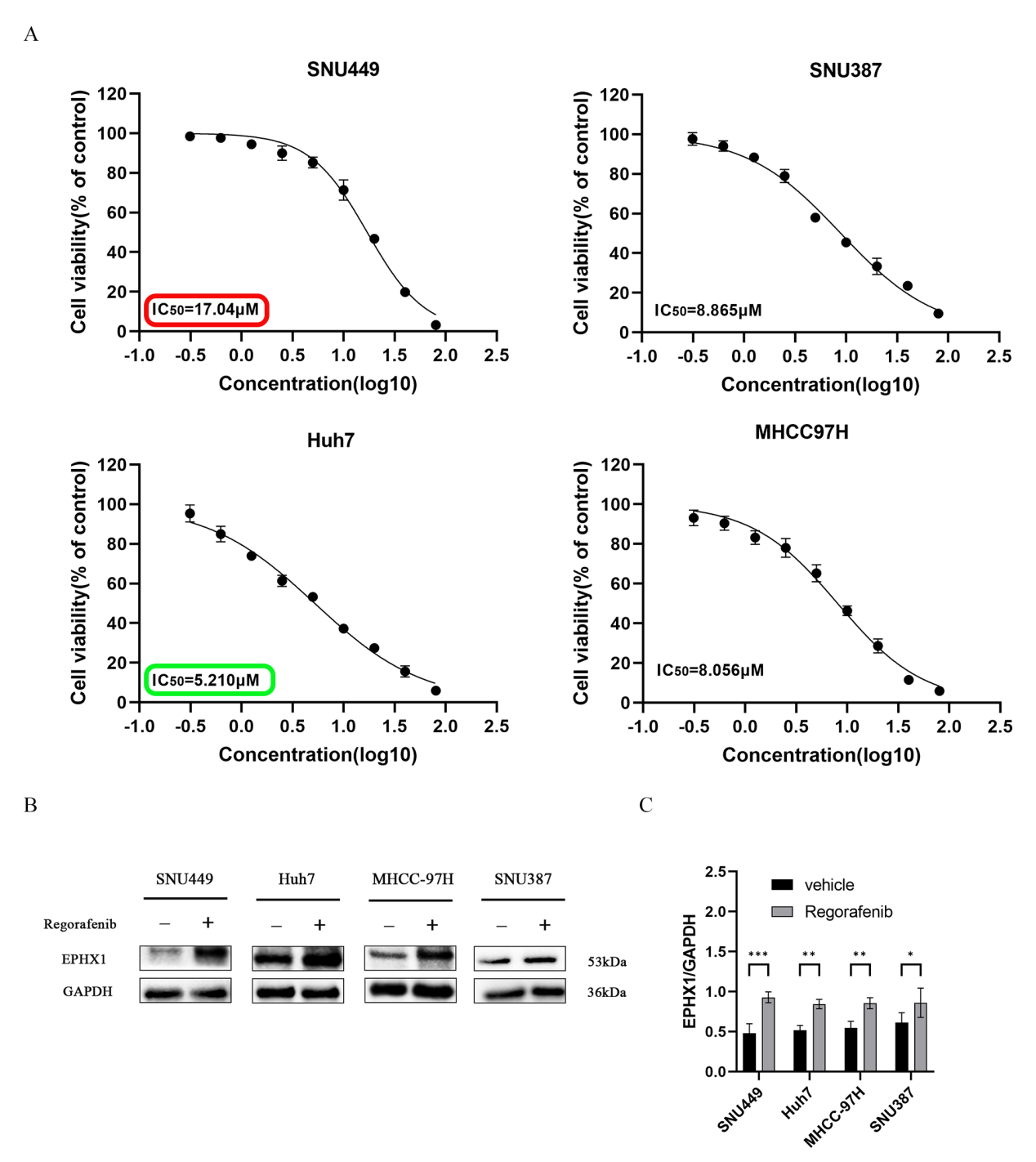


Supplementary Figure 1: **Induction of Regorafenib resistance by EPHX1 in hepatocarcinoma cell lines**. (A) The IC50 values of Regorafenib were detected by CCK-8 assays in four HCC cell lines. (B) Western blot analysis showed the levels of EPHX1 in four HCC cell lines after Regorafenib treatment. * *p* < 0.05，** *p* < 0.01，*** *p* < 0.001.
