# Supplementary material for: EPHX1 enhances drug resistance to regorafenib by activating the JAK/STAT signaling pathway in hepatocellular carcinoma cell lines
Source: Hereditas. 2025 Jul 31;162:148. doi: 10.1186/s41065-025-00517-1 (PMC12315303; doi:10.1186/s41065-025-00517-1)
Supplement: Supplementary file 3 — Supplementary Material 3: Supplementary Figure 2: Induction of Regorafenib resistance by EPHX1 in hepatocarcinoma cell lines. (A) Transfection efficiency and mRNA expression level and protein expression level of EPHX1 of knock-down in SNU449. (B) Knock-down in Huh7. (C) Overexpression in SNU449. (D) Overexpression in Huh7. [file 41065_2025_517_MOESM3_ESM.docx]

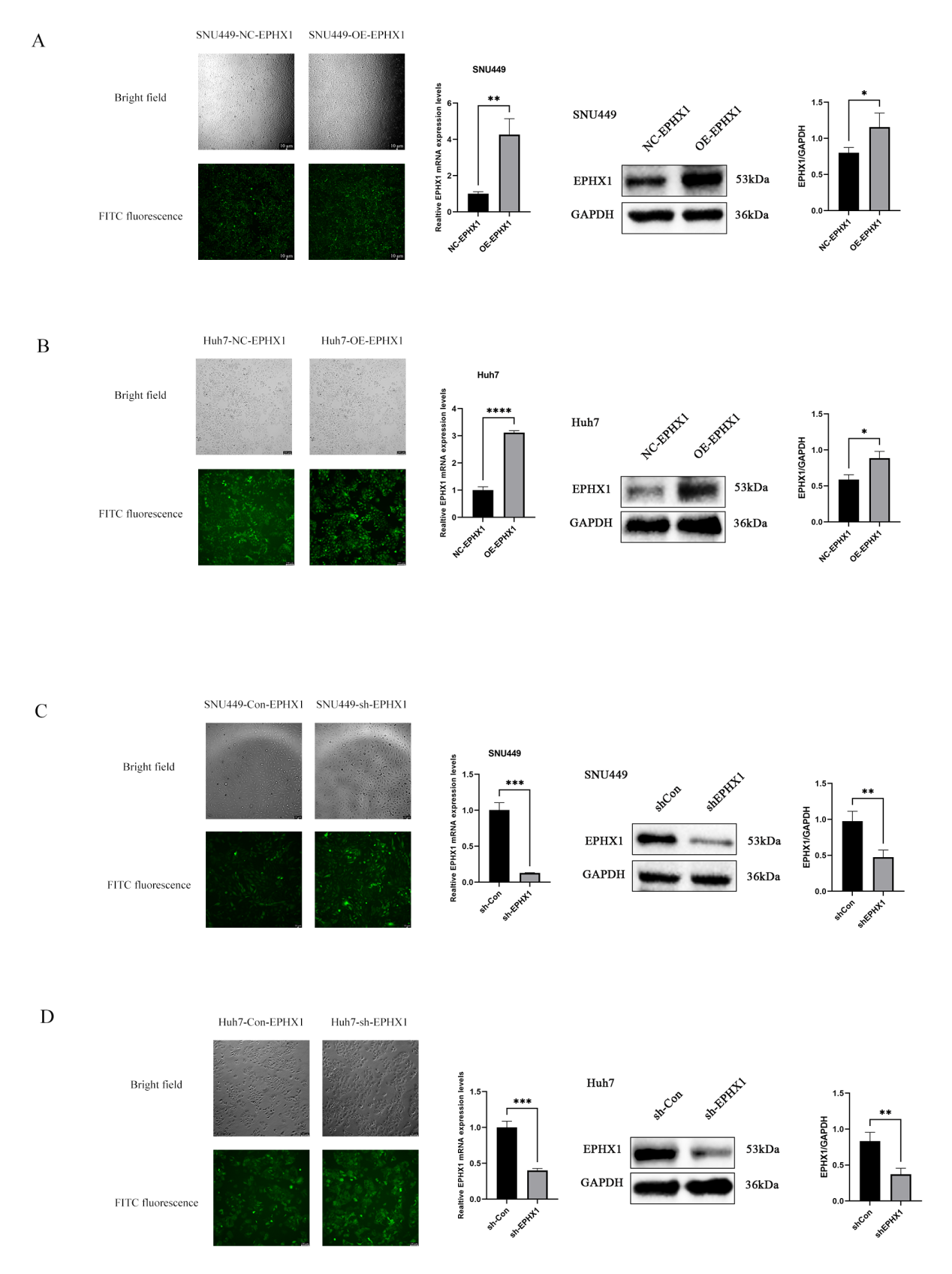


Supplementary Figure 2: **Induction of Regorafenib resistance by EPHX1 in hepatocarcinoma cell lines**. (A) Transfection efficiency and mRNA expression level and protein expression level of EPHX1 of knock-down in SNU449. (B) Knock-down in Huh7. (C) Overexpression in SNU449. (D) Overexpression in Huh7.
